# Vacuolin-1 inhibits endosomal trafficking and metastasis via CapZβ

**DOI:** 10.1038/s41388-021-01662-3

**Published:** 2021-02-09

**Authors:** Zuodong Ye, Dawei Wang, Yingying Lu, Yunjiao He, Jingting Yu, Wenjie Wei, Chang Chen, Rui Wang, Liang Zhang, Liangren Zhang, Minh T. N. Le, William C. Cho, Mengsu Yang, Hongmin Zhang, Jianbo Yue

**Affiliations:** 1 City University of Hong Kong Shenzhen Research Institute, Shenzhen, China; 2 Department of Biomedical Sciences, City University of Hong Kong, Hong Kong, China; 3 Department of Biology, Guangdong Provincial Key Laboratory of Cell Microenvironment and Disease Research and Shenzhen Key Laboratory of Cell Microenvironment, Southern University of Science and Technology, Shenzhen, 518055 China; 4 Research Core Facilities, South University of Science and Technology of China, Shenzhen, 518052 China; 5 State Key Laboratory of Natural and Biomimetic Drugs, School of Pharmaceutical Sciences, Peking University, 100191 Beijing, China; 6 Department of Pharmacology, Yong Loo Lin School of Medicine, National University of Singapore, Singapore, Singapore; 7 Department of Clinical Oncology, Queen Elizabeth Hospital, 30 Gascoigne Road, Kowloon, Hong Kong, China; 8 City University of Hong Kong Chengdu Research Institute, Chengdu, China

**Keywords:** Breast cancer, Drug development

## Abstract

Metastasis is the fundamental cause of cancer mortality, but there are still very few anti-metastatic drugs available. Endosomal trafficking has been implicated in tumor metastasis, and we have previously found that small chemical vacuolin-1 (V1) potently inhibits autophagosome-lysosome fusion and general endosomal-lysosomal degradation. Here, we assessed the anti-metastatic activity of V1 both in vitro and in vivo. V1 significantly inhibits colony formation, migration, and invasion of various cancer cells in vitro. It also compromises the assembly-disassembly dynamics of focal adhesions (FAs) by inhibiting the recycling and degradation of integrins. In various experimental or transgenic mouse models, V1 significantly suppresses the metastasis and/or tumor growth of breast cancer or melanoma. We further identified capping protein Zβ (CapZβ) as a V1 binding protein and showed that it is required for the V1-mediated inhibition of migration and metastasis of cancer cells. Collectively, our results indicate that V1 targets CapZβ to inhibit endosomal trafficking and metastasis.

## Introduction

One of the most challenging questions in cancer treatment is tumor metastasis [1, 2]. Metastasis refers to the spread of a tumor from its primary site to distant tissues or organs in the body [3]. Metastasis is a complex multistep process, involving cancer cell mobilization, invasion, intravasation, circulation in the blood vessels, extravasation, and metastatic colonization [4]. Although metastasis accounts for 90% of cancer mortality, anti-metastatic drugs are absent from the current anticancer arsenal, which is largely made up of anti-proliferation pathway drugs for tumor shrinkage [5, 6]. To fight cancer more effectively, a more in-depth understanding of the biology of metastases and the development of novel therapeutics that will combat the metastatic process are needed [6]. Endosomal trafficking has been reported to play a role in carcinogenesis, especially metastasis [7, 8].

Endocytosis is an evolutionarily conserved cellular process, which plays an important role in a wide variety of physiological functions; from development and immunity to neurotransmission. The dysregulation of endocytosis has been implicated in various human diseases, including autoimmune diseases, neurodegeneration, diabetes, and cancer [9–11]. Extracellular molecules or membrane proteins were internalized via clathrin-dependent/independent vesicles, which were subjected to various homotypic fusion events to form early endosomes. These comprise the primary sorting hub for determining the fate of these internalized materials. For example, important receptors can be either recycled back from the early endosomes to the plasma membrane or delivered to late endosomes, which then fuse with lysosomes for degradation. The maturation, sorting, and trafficking events of these vesicles are all tightly controlled by RAB small GTPases (RABs), membrane tethering complexes, SNAREs, and sorting nexin family proteins, as well as phosphatidylinositol phospholipids (PIPs) and their catalyzing enzymes [12–15]. The interplay among these complexes in regulating endosomal trafficking remains to be elucidated [14, 16]. Among the signaling molecules, the recruitment, activation, and inactivation of different RABs, e.g., RAB5, play an essential role in controlling the identity and maturation of endosomes [17–19].

Endosomal trafficking supplies the components of the plasma membrane of the cells, which is important to establish the polarity of cells and promote cell migration [9, 10, 20]. Dysregulation of specific trafficking regulators has been linking to all stages of carcinogenesis, from initial transformation to late invasion and metastasis [21, 22]. The vesicle trafficking in the neoplastic cells is essential for the membrane dynamics changes required for metastasis [7]. For example, integrins at the plasma membrane have been shown to turnover quickly from cell membranes through endocytosis, and the flux of integrins is positively correlated with cell migration and tumor metastasis [23, 24]. Yet, few specific drugs on endosomal trafficking have been identified thus far [7]. Elucidating the molecular mechanisms underlying endosomal trafficking should enable the development of specific drugs targeting this pathway to treat cancers, especially metastasis [25].

CapZβ forms a heterodimeric complex with CapZα, and this CapZβ-CapZα heterodimer, called capping protein (CP) or capping protein Z (CapZ), binds to the barbed ends of actin filaments to prevent further addition or loss of actin monomers [26]. CapZ has been shown to participate in a variety of cellular processes such as cell morphology, differentiation, and neural crest migration [26, 27]. However, CapZβ has recently been reported to regulate spindle assembly during mitosis independent of actin assembly [28]. In addition, CapZβ was found in the WASH complex on endosomes as revealed by a proteomics study, but its role in endosomal trafficking remains unclear [29].

By combining high-content fluorescence image-based drug screening, virtual drug screening, and chemical synthesis, we have identified several 6-morpholino-1,3,5-triazine derivatives as endosomal trafficking inhibitors, including vacuolin-1 (V1) [30, 31]. V1 was originally found to induce homotypic fusion of endosomes or lysosomes, thereby forming large vacuoles [32–34]. We further demonstrated that V1 blocks endosome maturation by activating RAB5, and this subsequently compromises the biogenesis and function of lysosomes, including autophagosomal-lysosomal fusion and endosomal-lysosomal degradation [31]. Here, we showed that V1 is a potent anti-metastatic drug, but more importantly, we demonstrated that this drug inhibits metastasis by inhibiting endocytosis as it over-activates RAB5 by targeting CapZβ.

## Results

### V1 potently inhibits the migration, invasion, and single colony formation of cancer cells in vitro

**Fig. 1:**
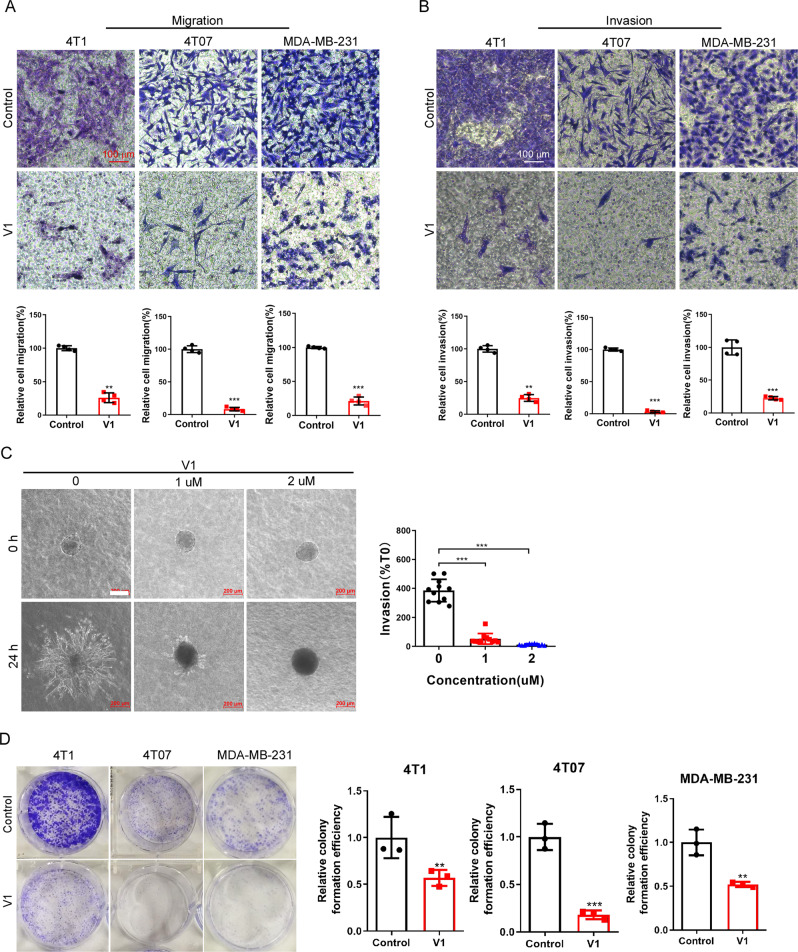
V1 inhibits the migration, invasion and colony formation of mammary carcinoma in vitro. (**A**) Migration and (**B**) invasion assays with 4T1, 4T07 and MDA-MB-231 cells. Cells were placed into the upper chamber of either a transwell plate (**A**) or an invasion chamber coated with Matrigel (**B**) in the absence (control) or presence of V1 (1 μM). After 18 h, the cells in the lower chamber were stained with crystal violet and quantified. Scale bar is 100 μm. **C** 3D matrix invasion assay for 4T1 cells. Scale bar is 200 μm. **D** V1 (1 μM) significantly inhibited the colony formation of 4T1, 4T07 and MDA-MB-231 cells. The graphs represented data from three independent experiments, and data quantifications were expressed as mean ± s.e.m. *P < 0.05, **P < 0.01, ***P < 0.001.

Since impaired endosomal trafficking has been implicated in the carcinogenesis, e.g., metastasis, of malignant tumors [21, 22], we investigated the effect of V1, an inhibitor of endosomal trafficking [30, 31], on the viability, proliferation, and migration of several human or mouse cancer cell lines, e.g., 4T1, MDA-MB-231, 4T07, etc. Although treatment of 4T1 or MDA-MB-231 cells with different concentrations of V1 exhibited little effects on cell viability (Fig. S1A), V1 at concentration >1 μM started to inhibit cell proliferation after 48 h treatment (Fig. S1B). Similar results have also been observed in HeLa, PC3, MCF-7, A549, and HepG2 cells [31]. Since the effective concentration of V1 to inhibit endolysosomal trafficking is around 1 μM [31] and treatment of cells with V1 at this concentration for 24 h had little effects on cell proliferation (Fig. S1B), in the following in vitro migration and invasion experiments, we mainly treated cells with V1 at 1 μM to exclude out the effects of cell proliferation on migration or invasion. We showed that V1 (1 μM) significantly inhibited the migration and invasion of cancer cells, as shown by the transwell migration/invasion assay and wound-healing assay (Figs. 1A, B and S1C, D). In contrast, chloroquine (CQ), which blocks autophagosome-lysosome fusion and endolysosomal degradation [31], at similar concentrations had a far less marked effect on cell migration (Fig. S1E). Moreover, V1 significantly inhibited the invasion of 4T1 mammary carcinoma in a 3D matrix invasion assay (Fig. 1C). As cancer cell motility is predictive of metastatic potential, we performed live-cell mobility tracking assay and confirmed that the V1 significantly inhibited the speed and distance of cell movement (Fig. S1F). In summary, these data indicate that V1 exhibits anti-migration activity in vitro.

In addition, we assessed the effects of V1 on the colony-formation ability of single cancer cells [35]. We found that V1 significantly inhibited the colony formation ability of various cancer cells, e.g., 4T1, 4T07, MDA-MB-231, A549, CNE-1, HeLa, and human lung cancer stem cells (LCSCs) (Figs. 1D and S1G). The anti-proliferation activity of V1 after the prolonged treatment of cells might contribute to its ability to inhibit colony formation of cancer cells. Taken together, these results suggest that V1 might be an effective anticancer drug.

### V1 potently inhibits the recycling of integrins and the dynamics of focal adhesions

**Fig. 2:**
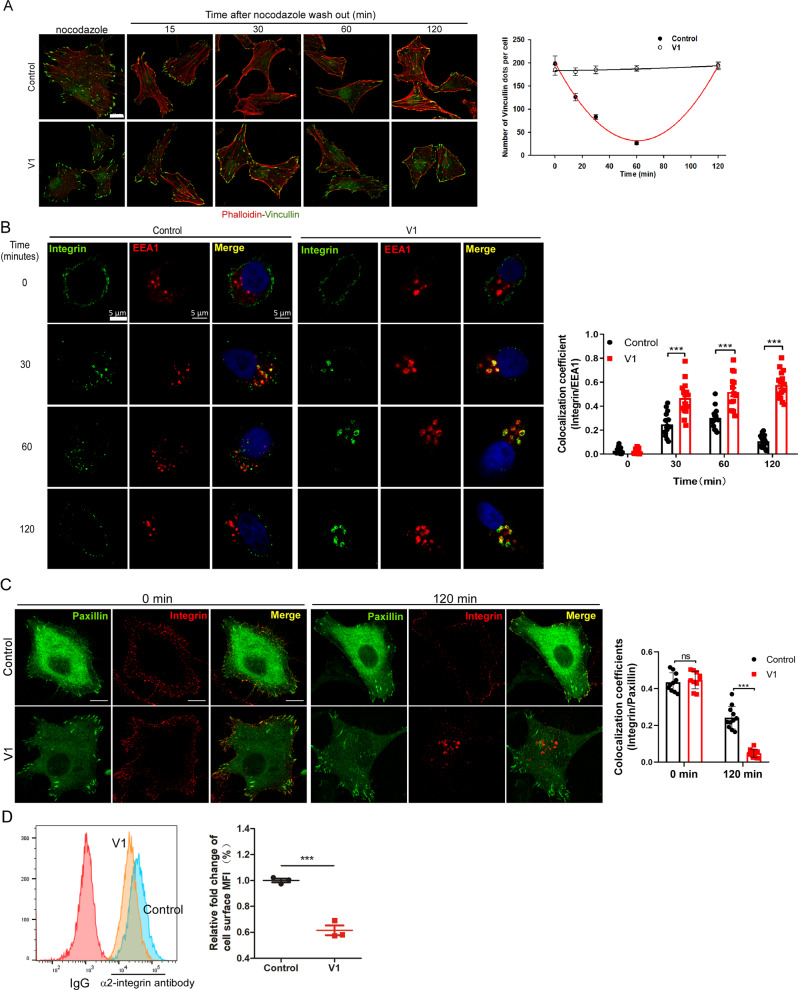
V1 inhibits the recycling of integrins. **A** HeLa cells were plated on coverslips in 24-well plates and treated with nocodazole (NOC) in the absence (control) or presence of V1 (1 μM). At indicated time points after removing NOC, the cells were stained with an anti-vinculin antibody and counterstained with phalloidin, after which the vinculin puncta were quantified. Scale bar is 10 μm. **B** HeLa cells were plated on coverslips in 24-well plates and treated with or without V1 (1 μM), followed by incubation with an anti-α2 integrin antibody on ice for 1.5 h. At the indicated time points after release from cold arrest, the cells were fixed and stained with an anti-EEA1 antibody. Scale bar is 5 μm. **C** HeLa cells were transfected with paxillin-GFP, and stained with α2 integrin antibody (red) on ice for 1.5 h. 2 h after release from cold arrest, the cells were fixed and imaged. Scale bar is 5 μm. **D** Flow cytometry-based quantification of plasma membrane levels of α2 integrin in HeLa cells treated with or without V1 (1 μM) for 24 h. The graphs represented data from three independent experiments, and data quantifications were expressed as mean ± s.e.m. *P < 0.05, **P < 0.01, ***P < 0.001.

We subsequently investigated the mechanisms underlying the anti-migration activity of V1. Since the dynamic action of focal adhesions (FAs) is a key process during cell migration [36, 37], we first assessed whether V1 treatment affects the spatiotemporal regulation of FA dynamics. Treatment of cells with nocodazole (NOC) depolymerized the microtubules (Fig. S2A) and stabilized the formation of FAs, as shown by the punctate staining pattern of Vinculin, a cytoplasmic actin-binding protein enriched in FAs (Fig. 2A). The removal of NOC led to microtubule regrowth (Fig. S2A), and this resulted in a decrease in the number of FAs in a time-dependent manner, as manifested by the gradual disappearance of Vinculin puncta from 15 min to 60 min. Thereafter, FAs were reassembled, as shown by the reappearance of vinculin puncta after 120 min following NOC removal (top panel in Fig. 2A). However, V1 treatment markedly inhibited FA disassembly, as shown by the continuing localization of Vinculin puncta following NOC removal (bottom panel in Fig. 2A). Similarly, the assembly-disassembly dynamics of FAs were observed in control GFP-Paxillin-expressing HeLa cells, but not in GFP-Paxillin-expressing cells treated with V1, by time-lapse confocal imaging (Fig. S2B, and Videos S1 and S2). These data demonstrate that V1 compromises the assembly-disassembly dynamics of FAs, which might lead to the inhibition of migration and invasion of tumor cells.

The turnover and recycling of different integrins heterodimers is the key component involving in tumor invasion and metastasis. During cell migration, integrin trafficking is important for the dynamic assembly/disassembly of the FA complex [38, 39]. Thus, we examined whether V1 treatment might affect the traffic of integrins. Cells were first incubated with an α2-integrin antibody on ice for 90 min, and the internalization of the integrin-antibody complex was then initiated at 37 °C [40]. In control cells, within ~30–60 min, the integrin-antibody complex had re-localized from the cell membrane to the early endosomes, as manifested by the colocalization of integrin and EEA1, an early endosomal marker (Figs. 2B and S2C). The majority of the internalized integrins were recycled back to the cell membrane after ~2 h (Figs. 2B, C, and S2C, D), and others might be sent to lysosomes, as shown by the colocalization of integrin and LAMP1, a late endosome/lysosome marker (Fig. S2D). In contrast, following V1 treatment, the integrin-antibody complex was trapped in early endosomes, and it failed to be either recycled back to the cell membrane or sent to lysosomes (Figs. 2B, C, and S2C, D). Thus, these data indicate that V1 inhibits the dynamics of integrin traffic. Notably, the recycled integrin could re-colocalize with FAs in control cells (top right panel in Fig. 2C), not in V1-treated cells (bottom right panel in Fig. 2C). As expected, V1 treatment markedly reduced cell-surface integrin levels (Fig. 2D) but did not affect the total integrin levels (Fig. S2E). These data suggest that the blockage of the integrin traffic process by V1 may contribute to the defects of FA turnover and recycling in V1-treated cells, which leads to the inhibition of migration and invasion of tumor cells.

### V1 inhibits both tumor growth and metastasis of mammary adenocarcinomas in MMTV-PyMT transgenic mice

**Fig. 3:**
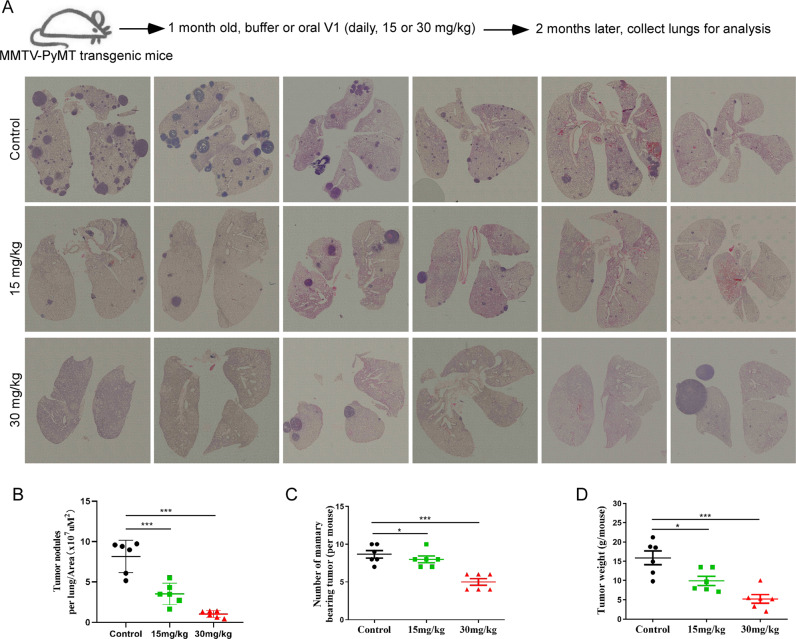
V1 significantly inhibits mouse mammary carcinoma metastasis in the MMTV-PyMT transgenic mouse model. One-month-old female MMTV-PyMT transgenic mice were randomly divided into three groups (*n* = 6 per group) and treated with either buffer or V1 (at either 15 or 30 mg/kg, daily) via oral gavage for 2 months. At the end of the experiment, the lungs in each group were collected and subjected to H&E staining (**A**), and the number of tumor nodules was normalized to the area of the lung, respectively(**B**), the number of mammary bearing tumors (**C**) and the weight of mammary gland tumors (**D**) were measured and quantified. Total of 4-5 sections per sample(total 6 mice per group) in different positions were used for quantification. Data quantifications were analyzed using ANOVA test and expressed as mean ± s.e.m, *p < 0.05, **p < 0.01, ***p < 0.001.

Since V1 potently inhibited the migration, invasion, proliferation, and single colony formation of cancer cells in vitro (Figs. 1 and S1), we examined the in vivo anticarcinogenic activities of V1 in the MMTV-PyMT transgenic mice. In female MMTV-PyMT mice, the mammary gland-specific expression of PyMT results in the development of mammary adenocarcinomas and metastatic lesions in the lymph nodes and lungs [41]. We showed that V1 treatment (oral delivery of 15 mg/kg or 30 mg/kg per day) significantly decreased the number of tumor nodules in the lungs (Fig. 3A, B), the number of the mammary bearing tumor (Fig. 3C), and the weight of mammary tumors in a concentration-dependent manner (Fig. 3D). Similar results have been observed following intraperitoneal (IP) delivery of V1 (20 mg/kg, daily) in this transgenic mouse model (Fig. S3A–D). Taken together, these results demonstrate that V1 potently inhibits both tumor growth and metastasis of mouse MMTV-PyMT mammary carcinoma.

### V1 inhibits the metastasis of mammary carcinoma in an experimental mouse model

**Fig. 4:**
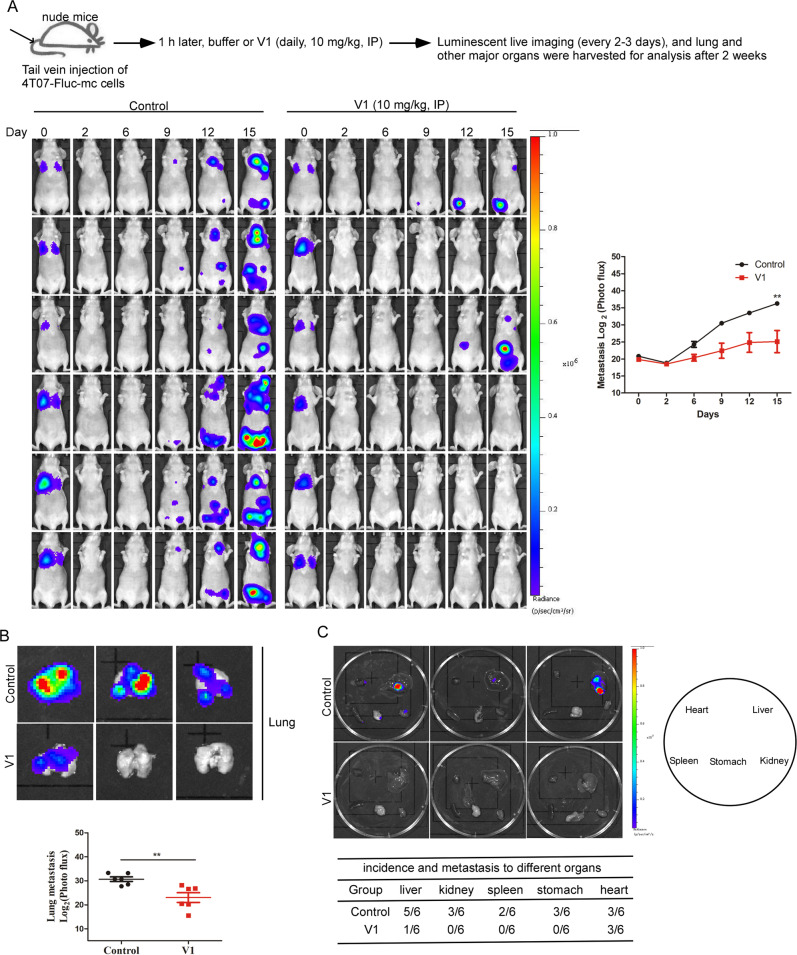
V1 significantly inhibits tumor metastasis in an experimental mouse model. Fluc-mCherry-expressing 4T07 cells were injected into the tail vein of nude mice. 1 h after injection, the mice were randomly divided into two groups (*n* = 6 per group) and treated with either buffer or V1 (10 mg/kg, IP, daily) for 2 weeks. During the course of the experiment, the mice were imaged every 3-4 days, and the luminescent signals were quantified (**A**). At the end of experiment, the lungs (**B**) and other major organs (**C**) in each group were collected and imaged, and the luminescent signals were quantified. Data shown are mean ± s.e.m, *p < 0.05, **p < 0.01, ***p < 0.001.

The tail vein injection model is commonly used to study the late stages (extravasation and metastatic colonization) of metastasis [42–44]. Thus, we set out to determine the anti-metastasis activity of V1 in an experimental mouse model, which was established by tail vein injection of 4T07 mouse mammary carcinoma expressing luciferase and mCherry into nude mice. At 1 h following 4T07 injection, the mice were then treated with or without V1 via IP injection (10 mg/kg/per day) for two weeks. In the V1-treated mice, far weaker luminescent signals were spread over a smaller area than that in the control mice (Fig. 4A). The luminescent imaging of isolated major organs also verified that the organs in the control groups exhibited strong luminescent signals, whereas those in the V1-treated groups showed much weaker signals (Fig. 4B, C). Besides IP injection of V1, oral delivery of V1 (15 or 30 mg/kg, daily) also significantly inhibited luminescent signals of the lungs as well (Fig. S4A–D). These results indicate that V1 significantly inhibits lung colonization.

Similarly, after tail vein injection of CA1a-Fluc-mc human breast cancer cells into NSG mice, the luminescent signals of the lungs in the mice treated by V1 (10 mg/kg/per day, IP) were weaker when compared to control mice treated with vehicle (Fig. S5A, B). Likewise, after tail vein injection of A375 human melanoma cells into NSG mice, the number of the tumor nodules found in the lungs of the mice treated with V1 (10 mg/kg/per day, IP) were fewer than that in the control mice (Fig. S5C). We speculate that the ability of V1 to inhibit the metastasis of cancer cells in this tail vein injection mouse model is likely due to its ability to inhibit the colony formation and/or proliferation of cancer cells (Figs. 1D and S1B). Nevertheless, these results indicate that V1 exhibits anti-metastatic activity in vivo.

### V1 inhibits the metastasis of mouse mammary carcinoma in an orthotopic metastatic mouse model

**Fig. 5:**
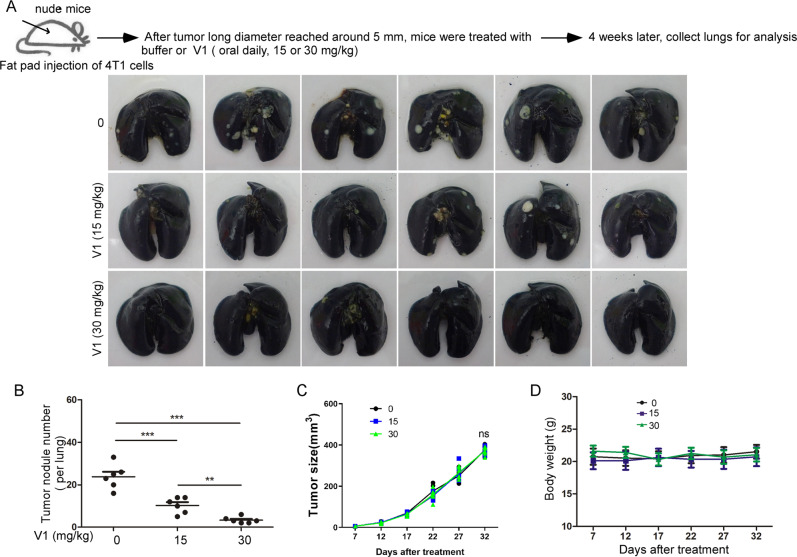
V1 significantly inhibits mouse mammary carcinoma metastasis in an orthotopic metastatic mouse model. 4T1 cells were injected into the fat pads of female nude mice. After tumors had grown to ~5 mm in long diameter, the mice were randomly divided into three groups (*n* = 6 per group) and treated for 4 weeks with either buffer or V1 (at either 15 or 30 mg/kg, daily) via oral gavage. At the end of each experiment, the lungs in each of the three groups were collected and stained with ink (**A**), after which the number of tumor nodules in each lungs was quantified (**B**). During the course of the experiment, the tumor size (**C**) and mouse body weight (**D**) were measured every 5 days. Data quantifications were analyzed using ANOVA test and expressed as mean ± s.e.m, *p < 0.05, **p < 0.01, ***p < 0.001.

To further assess the anti-metastasis ability of V1 from the primary tumor to distant organ site, we next established an orthotopic metastatic breast cancer mouse model by injection of 4T1 mouse mammary carcinoma in the mammary fat pad of nude mice (Fig. 5A). After tumors were palpable (around day 9), mice were orally treated with or without V1 (15 or 30 mg/ml) every day. As expected, V1 significantly inhibited the metastasis of 4T1 cells in the nude mice in a concentration-dependent manner, as demonstrated by fewer tumor nodules in the lungs of the V1-treated groups when compared with the control groups (Fig. 5A, B). Notably, V1 did not affect the growth of primary tumors (Fig. 5C), or the mouse weight (Fig. 5D). Likewise, the IP injection of V1 showed a similar anti-metastatic effect as the oral delivery route (Fig. S6). In addition, metastatic tumor nodules were found in the liver of control animals, but not in those treated with V1 (Fig. S6E). Since V1 had no effects on the growth of the primary tumors in the nude mice, the ability of V1 to inhibit the metastasis of cancer cells from the primary tumor is likely due to its ability to inhibit the migration, invasion, and proliferation of cancer cells. Nevertheless, these results demonstrate that V1 could efficiently suppress cancer metastasis from the primary tumor to distant organs.

### V1 enhances the anti-metastatic effects of chemotherapy

**Fig. 6:**
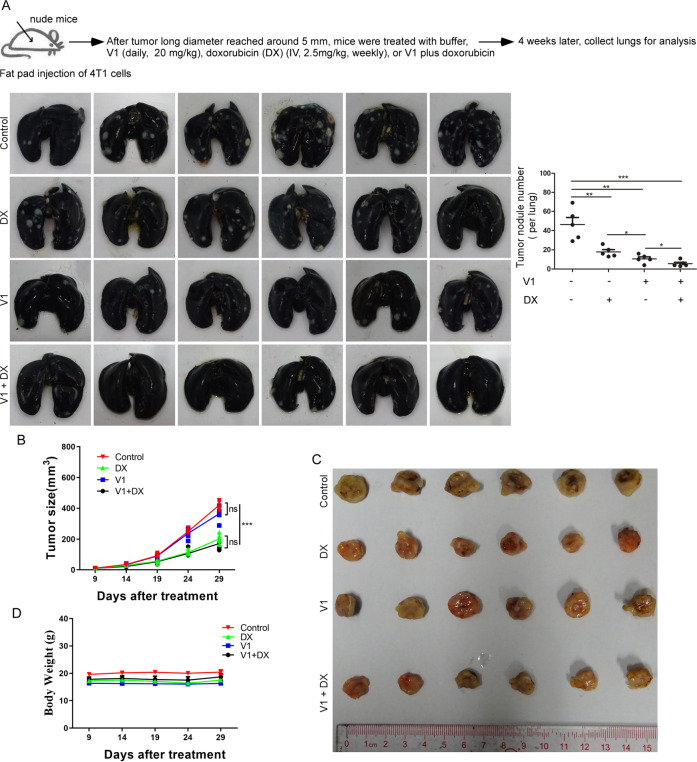
V1 in combination with doxorubicin significantly inhibits metastasis and breast tumor growth in an orthotopic metastatic mouse mammary carcinoma model. 4T1 cells were injected into the fat pads of female nude mice. After tumors had grown to ~5 mm in long diameter, the mice were randomly divided into four groups (*n* = 6 per group) and treated with buffer, V1 (20 mg/kg, IP, daily), doxorubicin (2.5 mg/kg, IV, weekly), or V1 (20 mg/kg, IP, daily) plus doxorubicin (2.5 mg/kg, IV, weekly) for 4 weeks. At the end of the experiment, the lungs in each group were collected and stained with ink, and the number of tumor nodules was quantified **(A)**. During the course of each experiment, the size of the tumor (**B** and **C**) and the body weight of the mice (**D**) were measured every five days. Data quantification were analyzed using ANOVA test and expressed as mean ± s.e.m, *p < 0.05, **p < 0.01, ***p < 0.001.

Since chemotherapy is the main treatment option for advanced or metastatic breast cancer patients, we assessed the combinatorial effects of V1 with doxorubicin [45], a chemotherapy drug, in our orthotopic metastatic breast cancer mouse model. Whereas doxorubicin significantly inhibited both tumor growth and metastasis, V1 did not affect tumor growth but had a more profound inhibitory effect on metastasis than doxorubicin (Fig. 6A–C). Interestingly, when V1 was used in combination with doxorubicin, there was no additional effect on the inhibition of tumor growth when compared with doxorubicin alone. However, the combination of V1 and doxorubicin significantly inhibited metastasis when compared with either of these drugs applied alone (Fig. 6A–C). Notably, neither of our treatment regimens had a marked effect on the body weight of the mice (Fig. 6D). In summary, these data indicate that V1, as an effective anti-metastatic drug, can be used in combination therapy with other therapeutics against cancer.

### V1 exhibits low acute and sub-chronic toxicity in mice

To investigate the in vivo toxicity of V1, We first performed a 30-day oral repeat dose toxicity study of V1 (30 mg/kg) in normal mice and found that no drug-induced deaths, no drug effects on body weight, no obvious changes in liver or kidney function test, and no signs of pathological changes in major organs after the repeated dose of V1 oral administration (Fig. S7). Likewise, the acute toxicity of oral delivery of V1 at a single dose of 1000 mg/kg showed that the mice were well tolerated this high dose, exhibiting no signs of toxicity in mice (Fig. S8). In summary, these data support that V1 is a potential oral anti-metastatic therapeutics with a good therapeutic window.

### V1 targets CapZβ to inhibit endosomal trafficking and metastasis

**Fig. 7:**
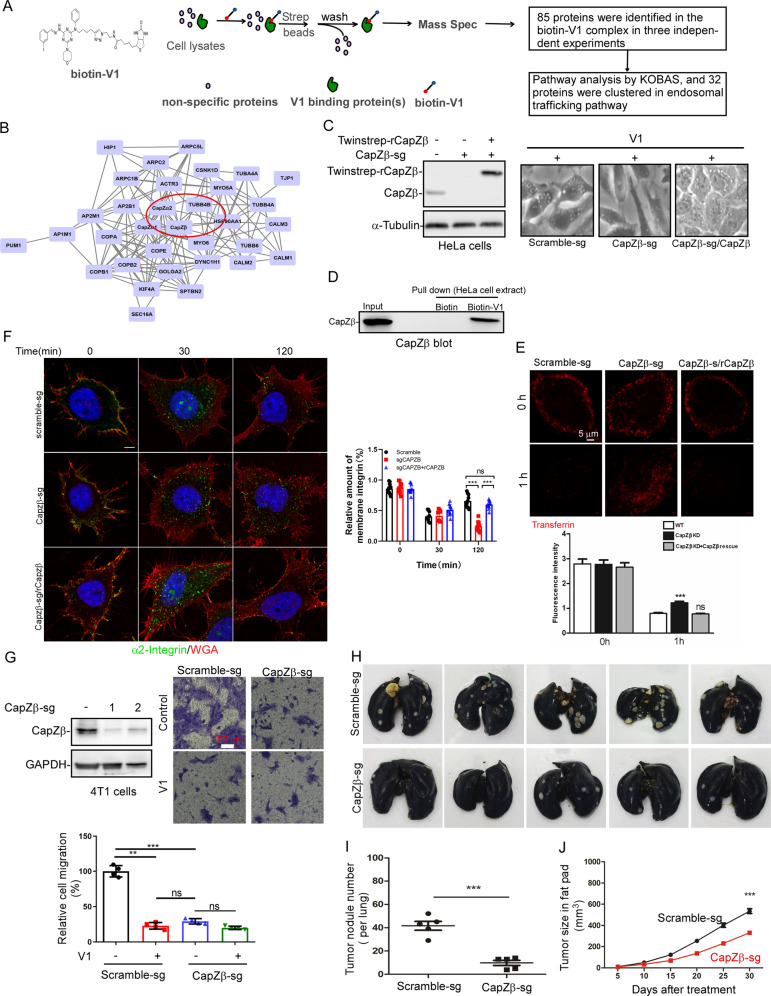
V1 targets CapZβ to inhibit endocytosis and metastasis. **A**, **B** HeLa cell lysates were incubated with biotin-V1, after which V1 binding proteins were pulled down using streptavidin beads and they were then analyzed via mass spectrometry (**A**). The identified V1 binding proteins were further analyzed with KOBAS, and 33 proteins, including CapZβ were shown to be clustered in the endosomal trafficking pathway (**B**). **C** When CapZβ was knocked out by CRISPR/Cas9 in HeLa cells, it abolished V1-induced large vacuoles, while addback of rCapZβ restored V1-induced large vacuoles. **D** The lysates prepared from HeLa cells treated with biotin or biotin-V1 were incubated with streptavidin beads, and the streptavidin pulldowns were subjected to CapZβ immunoblotting. **E**, **F** When CapZβ was knocked out by CRISPR/Cas9 in HeLa cells, it inhibited transferrin degradation (**E**) and integrin recycling (**F**). Cells were also stained with WGA (red) in **F**. These defects were restored by transfecting back CapZβ (rCapZβ). Scale bar is 5 μm. **G** CapZβ knockout inhibited the migration of 4T1 cells, but V1 treatment had no additive effect on the inhibition of migration of the CapZβ knockout cells. Scale bar is 100 μm. **H**–**J** Control or CapZβ-knockout 4T1 cells were injected into the fat pads of female nude mice. After one month, the lungs in each group were collected and stained with ink (**H**), and the number of tumor nodules in the lungs was quantified (**I**). During the course of these experiments, the tumor size was also measured every 5 days (**J**). Data quantifications were analyzed using ANOVA test and expressed as mean ± s.e.m, *p < 0.05, **p < 0.01, ***p < 0.001.

To identify the V1-binding proteins, we synthesized a biotin-V1 analog (biotin-V1) (Fig. S9A–C), and we found that similar to V1, biotin-V1 was able to: induce vacuoles (Fig. S9D); trigger the accumulation of LC3-II and p62 (Fig. S9E); inhibit transferrin degradation (Fig. S9F). Subsequently, we applied biotin-V1 to HeLa cell extracts, purified the V1-binding complex with streptavidin beads, and identified 85 potential V1-binding proteins by mass spectrometry (Fig. 7A). Among them, 32 proteins were clustered in the endocytosis and vesicle-mediated transport pathways using the KOBAS online software. Several hub proteins were identified via STRING online software, including GOLGA2, CapZβ, SPTBN2, and MYO5A (Figs. 7B and S9G). We subsequently knocked out each of these genes in HeLa cells and assessed whether the ability of V1 to induce vacuole formation or endocytosis arrest was affected. Interestingly, only knockout of CapZβ abolished the ability of V1 to induce vacuoles, and addback CapZβ (rCapZβ) restored V1-induced vacuoles (Fig. 7C). CapZβ (capping protein Zβ) is a canonical actin regulatory protein [26]. Indeed, streptavidin beads pulled down CapZβ from biotin-V1- (but not biotin-) treated HeLa cell extracts as shown by CapZβ immunoblot analysis (Fig. 7D). Likewise, biotin-V1 interacted with the recombinant CapZβ protein in vitro (Fig. S9H). Isothermal titration calorimetry (ITC) further confirmed the interaction between CapZβ and biotin-V1, and K_D_ between CapZβ and biotin-V1 is around 3.1 ± 0.8 μM (Fig. S9I), which is similar to the effective concentration of biotin-V1 to inhibit endocytosis (Fig. S9E, F). Taken together, these results indicate that CapZβ is a V1 binding protein.

We next assessed the role of CapZβ in the endocytosis process and found that CapZβ knockout significantly inhibited transferrin degradation, whereas addback rCapZβ to CapZβ knockout cells restored transferrin degradation (Fig. 7E). Likewise, CapZβ knockout abolished integrin recycling, and this was rescued by rCapZβ addback (Fig. 7F). We then examined the role of CapZβ in the migration of HeLa cells treated with or without V1. CapZβ knockout significantly inhibited cell migration, and treatment of CapZβ-knockout cells with V1 had no additional inhibitory effect on cell migration. rCapZβ addback rescued migration defects and restored cell sensitivity to V1-mediated migration inhibition (Fig. S9J). Consistently, CapZβ knockout in 4T1 cells also significantly inhibited migration, and treatment of CapZβ-knockout cells with V1 had no additional inhibitory effect on cell migration (Fig. 7G). These data suggest that CapZβ is an effector of V1 in endosomal trafficking and migration. We further studied the role of CapZβ in metastasis in our orthotopic cancer mouse model by injecting control or CapZβ-knockout 4T1 mammary carcinoma. CapZβ knockout significantly inhibited the metastasis of 4T1 cells, as manifested by the reduced number of tumor nodules per lung in mice implanted with CapZβ-knockout cells, when compared with mice implanted with control cells (Fig. 7H, I). This suggests that CapZβ is involved in tumor metastasis in vivo. Notably, the growth of tumors in mice implanted with CapZβ-knockout 4T1 cells was slower than that in mice implanted with control cells (Fig. 7J). The effect of CapZβ knockout on tumor growth is likely due to its canonical role in regulating actin polymerization besides endosomal trafficking. In summary, these results suggest that CapZβ mediates the ability of V1 to inhibit endocytosis, migration, and metastasis.

Interestingly, CapZβ was found to be one of the protein markers for breast cancer grading and staging [46], and V1 significantly inhibited the metastasis of triple-negative breast cancer (TNBC) cells, e.g., 4T1 and CA1a, in various experimental mouse models (Figs. 4, 5, S3 and S4). We, thus, analyzed CapZβ expression in TNBC via UALCAN, a web-portal for in-depth analyses of TCGA gene expression data [47] and datasets of GSE38959 and GSE53752 from the Gene Expression Omnibus (GEO) database (http://www.ncbi.nlm.nih.gov/geo), and found that CapZβ transcript in TNBC was indeed significantly increased as compared to the corresponding normal tissue (Fig. S10). Therefore, CapZβ might be a potential therapeutic target for metastasis.

### CapZβ is required for earlier endosome maturation

**Fig. 8:**
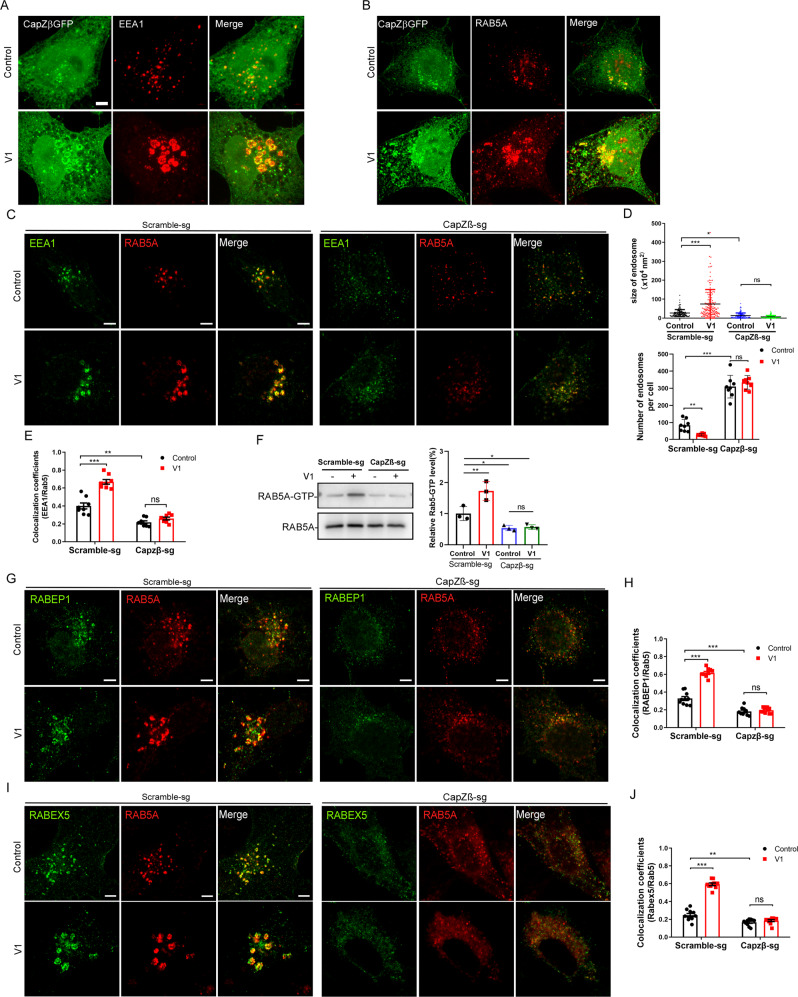
CapZβ is required for early endosome maturation. CapZβ-GFP expressing HeLa cells were either untreated (controls) or treated with V1 (1 μM) and then immunostained with an anti-EEA1 (**A**) or anti-RAB5 (**B**) antibody followed by confocal imaging. **C**, **E** Control or CapZβ-knockout HeLa cells treated with or without V1 (1 μM) were immunostained with the anti-EEA1 and anti-RAB5 antibody (**C**). The number and size of the early endosomes in control or CapZβ knockout cells were quantified (**D**). In addition, the colocalization coefficients of EEA1/RAB5 were quantified (**E**). **F** Active RAB5 in control or CapZβ knockout cells treated with or without V1 (1 μM) were examined with a GST–R5BD pulldown assay. **G, H** Control or CapZβ-knockout cells treated with or without V1 (1 μM) were immunostained with an anti-RAB5 or anti-RABEP1 antibody (**G**), after which the colocalization coefficients of RABEP1/RAB5 were quantified (**H**). **I, J** Control or CapZβ-knockout cells treated with or without V1 (1 μM) were immunostained with an anti-RAB5 or anti-RABEX5 antibody (**I**), after which the colocalization coefficients of RABEX5/RAB5 were quantified (**J**). Data quantifications were analyzed using ANOVA test and expressed as mean ± s.e.m, *p < 0.05, **p < 0.01, ***p < 0.001.

To dissect how V1 regulates CapZβ to inhibit endolysosomal trafficking, we first examined the subcellular localization of CapZβ. Our data showed that in CapZβ-GFP-expressing cells, CapZβ was localized both diffusely and in puncta. Interestingly, some of the CapZβ puncta appeared to be organized into vesicular structures and were strongly associated with the early endosome markers, EEA1 and RAB5 in the control cells (upper panels in Fig. 8A, B. Furthermore, in V1-treated cells, CapZβ puncta are strikingly accumulated at the surface of the enlarged endosomes (bottom panels in Fig. 8A, B). These data suggest that CapZβ is associated with early endosomes. We next examined whether the association of CapZβ with endosomes is required for the maturation of early endosomes. The size of the early endosomes was much lower, whereas the number of endosomes was significantly higher in the CapZβ-knockout cells than in the control cells treated with or without V1 (Fig. 8C, D). This suggests that the fusion of small early endosomes in the CapZβ knockout cells is blocked or inhibited. Notably, it has been previously reported that the expression of a constitutively active (CA) RAB5 mutant (RAB5-Q78L) induces the homotypic fusion of the early endosomes, but blocks the early to late endosome transition, manifested by increased size but decreased the number of early endosomes in RAB5-Q78L-expressing cells [48]. Therefore, we examined whether CapZβ knockout affects the RAB5 activity by a GST-R5BD pulldown assay, in which the RAB5-binding domain (R5BD) of the Rab5 effector RABEP1 specifically binds to GTP-bound Rab5 [49]. The result showed that V1 significantly increased RAB5A activity, while the level of RAB5-GTP was significantly lower in the CapZβ knockout cells than that in the control cells, and V1 treatment also failed to increase RAB5-GTP level in CapZβ-knockout cells (Fig. 8F), indicating that RAB5 is less active in the CapZβ knockout cells. We speculate that V1 might facilitate the recruitment or stabilization of CapZβ to the early endosomes to induce the activation of RAB5.

**Fig. 9:**
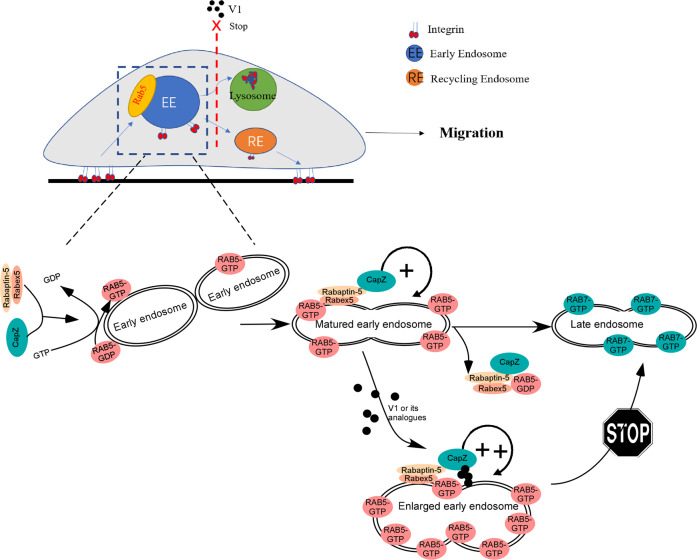
Model of how V1 targets CapZb to inhibit endosomal trafficking. CapZβ facilitates the recruitment of RABEX5, and RABEP1 on endosomes, thereby activating RAB5 during early endosome maturation. CapZβ on endosome initiates a positive feedback loop to increase RAB5 activity. V1 induces the accumulation of CapZβ on endosome, leading to the over-activation of RAB5 and enlarged early endosome, thus blocking the early-late endosome transition and causing defect of integrin trafficking and migration.

It has been reported that RAB5 on early endosomes recruits and activates more effectors, e.g, EEA1, RABEX5 (also called RABGEF1), RAPEP1 (also called Rabaptin-5), to endosomes, establishing a positive feedback loop to induce early endosome maturation [16]. We, therefore, examined the effects of CapZβ knockout on the association of RAB5 with its effectors during endocytosis. As expected, V1 treatment significantly induced the colocalization of RAB5 with EEA1 (Fig. 8C, E), RABEP1 (Fig. 8G, H), and RABEX5 (Fig. 8I, J). In contrast, CapZβ knockout abolished the V1-induced colocalization of RAB5 with EEA1, RABEP1, and RABEX5. Furthermore, even in the absence of V1, CapZβ knockout significantly inhibited the association of RAB5 with EEA1 (Fig. 8C, E), RABEP1 (Fig. 8G, H), or RABEX5 (Fig. 8I, J). Notably, the recruitment of RAB5 from the cytosol into endosomes and its subsequent activation promotes the maturation of early endosomes. Thereafter, RAB5 is inactivated, followed by the recruitment and activation of RAB7, which is essential for the transition from early endosomes to late endosomes [50]. Taken together, these results suggest that CapZβ facilitates the recruitment of EEA1, RABEX5, and RABEP1 on endosomes, thereby activating RAB5 during early endosome maturation. V1 treatment renders more CapZβ accumulated on early endosomes to over-activate RAB5, and this leads to enlarged early endosome and blockage of the early-late endosome transition (as the model shown in Fig. 9).

### RAB5 is required for V1-mediated metastasis inhibition

**Fig. 10:**
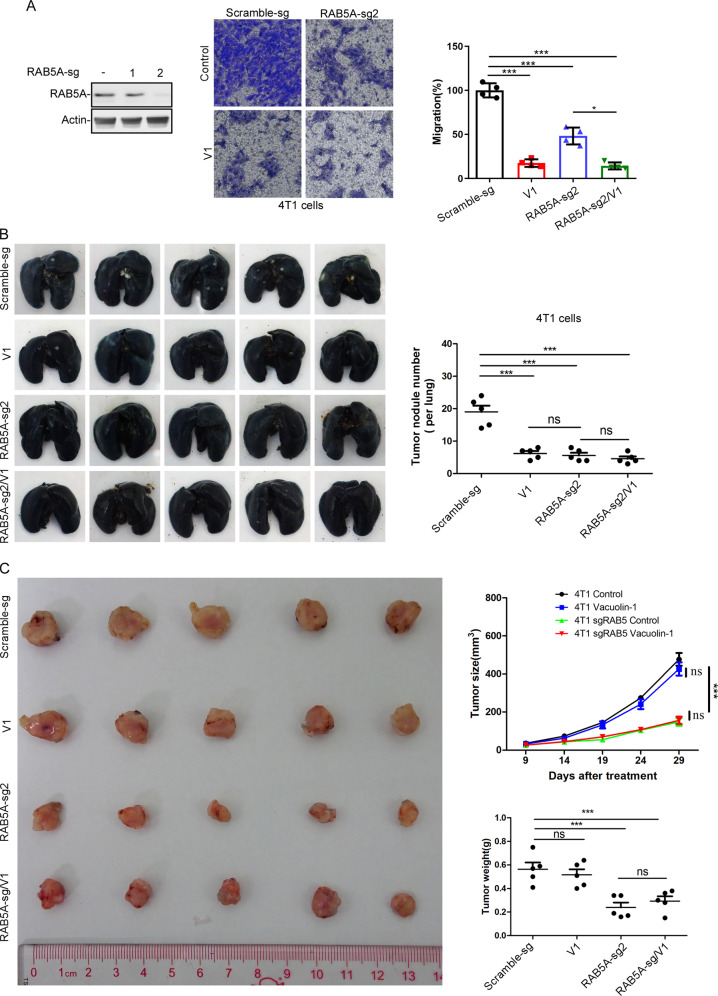
RAB5 is required for V1-mediated metastasis inhibition. **A** RAB5A knockout inhibited the migration of 4T1 cells, and V1 (1 μM) treatment further inhibited the migration of the RAB5A-knockout cells. **B, C** Control or RAB5A-knockout 4T1 cells were injected into the fat pads of female nude mice. After tumors had grown to ~5 mm in long diameter, the mice were randomly divided into four groups (*n* = 5 per group) and treated with buffer or V1 (30 mg/kg, oral, daily) for 4 weeks. At the end of the experiment, the lungs in each group were collected and stained with ink, and the number of tumor nodules was quantified **(B)**. During the course of the experiment, the size of the tumor was measured every five days, and the weight of the tumor was also measured at the end of each experiment (**C**). Data quantifications were analyzed using ANOVA test and expressed as mean ± s.e.m, *p < 0.05, **p < 0.01, ***p < 0.001.

To further demonstrate the role of endosomal trafficking in V1-mediated inhibition of migration and metastasis, we assessed the effects of RAB5 knockout on these two processes. RAB5, an essential player for the maturation of early endosomes, is involved in V1-mediated endosomal trafficking inhibition [31]. We found that RAB5A knockout in 4T1 cells significantly inhibited cell migration, yet V1 treatment of RAB5A-knockout cells further inhibited cell migration when compared to RAB5A-knockout cells alone (Fig. 10A). These results suggest that RAB5A is at least partially involved in V1-mediated cell migration inhibition. We then examined the role of RAB5 in metastasis in vivo. As shown in Fig. 10B, the number of tumor nodules per lung in mice implanted with RAB5A-knockout 4T1 cells was significantly lower than the mice implanted with control cells, which was similar to that in mice implanted with control cells treated with V1. In addition, treatment of the mice implanted with RAB5A-knockout cells with V1 failed to further decrease the number of tumor nodules per lung. However, the growth of primary tumors in mice implanted with RAB5A-knockout 4T1 cells was slower than the mice implanted with control cells, and V1 treatment failed to further slow the growth of RAB5A-deficient tumors (Fig. 10C). Since RAB5 plays a role in Ras, Rho, and other membrane receptors-mediated signaling events besides migration [51], we speculate that this might be one reason underlying the slower growth of the primary tumors in mice implanted with RAB5A-knockout cells. Nevertheless, these results, in which knockout of RAB5A or CapZβ (two proteins involving in the endocytosis) inhibited cell migration and cancer metastasis (Figs. 7 and 10), support the role of endosomal trafficking in V1-mediated migration and metastasis inhibition.

## Discussion

V1 was identified by us as being autophagy and endosomal trafficking inhibitor, which is more potent than well-established autophagy/endosomal trafficking inhibitors such as chloroquine [31]. It should be noted that over 50 human preclinical and clinical trials are ongoing to evaluate the anticancer effects of chloroquine or hydroxychloroquine [52]. Here, we found that V1, but not chloroquine, markedly inhibited the migration and invasion of cancer cells in vitro (Figs. 1 and S1). Moreover, V1 alone via different administration routes or in combination with other chemotherapeutic drugs significantly inhibited the metastasis of breast cancer or melanoma in various mouse models (Figs. 3–5 and S3–S6). Particularly in an orthotopic metastatic breast cancer mouse model, we found that V1 significantly inhibits breast cancer metastasis ability, but did not affect the growth of primary tumors (Figs. 5 and S6B), which indicate that the ability of V1 to inhibit migration and invasion contributes, at least partially, to its anti-metastasis activity. In the MMTV-PyMT mouse model, V1 treatment inhibited not only the metastasis but also the growth of the primary tumor (Fig. 3). The ability of V1 to inhibit proliferation, colony formation, migration, and/or other cellular processes might all contribute to the anti-metastatic activity of V1 in this transgenic mouse model. In addition, the low acute and sub-chronic toxicity in mice of V1 indicated that V1 is an effective anti-metastatic compound with the potential to be developed into a therapeutic drug(Figs. S7 and S8).

Although inhibitors of the α_v_β_3_ and α_v_β_5_ integrins targeting the ligand-binding sites showed promise in preclinical studies against metastasis, many of them failed in the clinical setting due to drug resistance or because of serious side effects [48, 53–55]. Considering that integrins play a role in almost every step in cancer metastasis, an alternative strategy to manipulate integrin function other than these antagonists should be examined as therapeutic against metastasis [56]. Here we found that V1 abolished the integrin trafficking by locking the internalized integrins in the early endosomes (Figs. 2B, C and S2C, D), and this is well correlated with the ability of V1 to stop the dynamic of FA turnover (Fig. 2A, C, and Videos S1 and S2) and to inhibit migration and invasion of tumor cells (Figs. 1 and S1). Therefore, V1 can be applied to manipulate the integrin function to inhibit migration and invasion of cancer cells.

Importantly, we identified CapZβ as being a V1 binding protein (Figs. 7 and S9). CapZβ knockout significantly inhibited the endosomal trafficking of transferrin and integrins and cell migration, while CapZβ addback restored transferrin degradation, integrins recycling and migration in CapZβ knockout cells. Moreover, CapZβ knockout significantly inhibited the metastasis of 4T1 cells in our orthotopic cancer mouse model (Fig. 7H). These results demonstrate that V1 targets CapZβ to inhibit endosomal trafficking, thereby suppressing cancer cell metastasis.

It is reported that RAB5 activation is essential for the biogenesis and maturation of early endosomes [50]. Notably, dominant-negative (DN) RAB5 mutant was shown to inhibit the maturation of the early endosomes, as revealed by the smaller endosomes [31, 57]. These data are therefore similar to our CapZβ knockout data (Fig. 8C, D). In our experiments, RAB5 was less active in the CapZβ-knockout cells when compared with the control cells treated with or without V1 (Fig. 8F). These data suggest that CapZβ is involved in the activation of RAB5 during endocytosis. However, CapZβ contains no conserved RAB-GEF domain, such as VPS9, DENN, or Sec2 [17, 19, 58–61], and lacks the longin-like fold to bind RABs [62, 63]. Thus, it is more likely that CapZβ activates RAB5 via other known RAB5 GEFs, such as RABEX5 [16, 17]. Indeed, we demonstrated that V1 induced the recruitment of RABEP1 and RABEX5 on the endosome (Fig. 8G–J). However, the interaction or association between RABEP1 or RABEX5 and RAB5 was significantly reduced in the CapZβ-knockout cells (Fig. 8G–J). These data suggest that CapZβ functions as a scaffold protein to carry RABEP1 or/and RABEX5 to RAB5-positive early endosomes and induce the production of RAB5-GTP. Furthermore, V1 may induce the accumulation of CapZβ on endosome, leading to the over-activation of RAB5 and thus blocking the early-late endosome transition (Fig. 9), which is similar to the phenotype of RAB5A constitutive active (RAB5A-CA) mutant [31]. Yet, the detailed molecular mechanism of CapZβ in endosomal trafficking remains to be determined.

## Supplementary information

supplemental material

control-paxillin dynamic

V1-paxillin dynamic

author change form 1

author change form 2
