# Supplementary material for: Vacuolin-1 inhibits endosomal trafficking and metastasis via CapZβ
Source: Oncogene. 2021 Feb 9;40(10):1775–91. doi: 10.1038/s41388-021-01662-3 (PMC7946642; doi:10.1038/s41388-021-01662-3)
Supplement: Supplementary file 1 — supplemental material [file 41388_2021_1662_MOESM1_ESM.docx]

**SUPPLEMENTAL MATERIALS AND DATA**

**MATERIALS AND METHODS**

*Cell culture-* HeLa, HEK 293T and MDA-MB-231 cells were obtained from ATCC. 4T07, 4T1, 4T07-Luciferase-mCherry cells, and MCF10CA1a-Luciferase-mCherry cells were kindly provided by Dr. Minh LE^60^. All the cells were maintained in DMEM (Invitrogen, 12800-017) containing 10% fetal bovine serum (Invitrogen, 10270-106) and 100 units/ml of penicillin/streptomycin (Invitrogen, 15140-122) at 5% CO_2_ and 37°C.

*Antibodies-* The LAMP1 and EEA1 rabbit monoclonal antibody were purchased from Cell Signaling Technologies. The GAPDH polyclonal antibody and CapZβ monoclonal antibody were ordered from Proteintech, whereas the alpha 2 integrin antibody (MCA2025) was purchased form Bio-Rad. The Alexa Fluor dye–conjugated secondary antibodies were purchased from Life Technologies.

*Cell migration assay-* Transwell migration assays were performed according to the manufacturer’s instructions with modifications. In brief, 1x10^5^ cells were resuspended in medium containing either DMSO or Vacuolin-1(1 μM) and then seeded on transwell filters (8 μm pore size; Corning 3422). After 18 h, cells on the upper surface of the filters were removed with cotton swabs, after which the cells on the lower surface were fixed in 4% paraformaldehyde and stained with 0.5% crystal violet. Images were taken under an inverted microscope (Nikon Eclipse TS100) and analyzed with the Image J software.

*Cell invasion assay-* Transwell invasion assays were performed according to the manufacturer’s instructions with a few modifications as follows. First, the transwell filters (8 μm pore size; Corning 3422) were precoated with Matrigel. Then, 1x10^5^ cells were resuspended in medium containing either DMSO or Vacuolin-1 (1 μM) and seeded onto these Matrigel-coated filters. After 18 h, the cells on the upper surface of each filter were removed with cotton swabs, and then the cells on the lower surface were fixed in 4% paraformaldehyde and stained with 0.5% crystal violet. Images were acquired and analyzed as described above.

*3D invasion assay-* 3D invasion assay was performed as described previously7. Briefly, 500 of cells single-cell suspension were used for hanging drop cultures to form tumor spheroid. After 72h, collect the spheroids and embedded into 3D Matrix mixture (basement membrane materials and type I collagen with ratio 1:1). Then, placed the viscous mixture into the centers of 24-well plate and keep the plate in 37 °C incubator and leave undisturbed for 30 min. Slowly add 1 ml of cell culture media DMSO or V1 into the 3D cultures and cultured at 37°C in a CO2 incubator. Monitoring Spheroid Invasion at 0h, 24h, 48h, and analyzed with Image J.

*Cell movement tracking assay***-** Cells were plated into 6-well tissue culture plates and allowed to adhere overnight. The following day, the cells were pretreated with either DMSO or V1 (1 μM) for 1 h, after which images to track the movement of cells were acquired every 30 min for 12 h under a Nikon Eclipse Ti-E Live cell imaging system and then analyzed with Image J.

*Focal adhesion dynamic assay-* Cells were seeded onto coverslips in 24-well plates. After incubation for 24h, the cells were treated with DMSO or Vacuolin-1 (1 μM) for 6 h, and then they were treated with nocodazole (10 μM) (Sigma) for a further 4 h. At the end of the nocodazole incubation, the medium was removed and replaced with completed medium containing either DMSO or V1. The cells were then collected at the time points indicated, and then fixed with 4% paraformaldehyde, before they were immunostained with an anti-vinculin antibody and counterstained with Texas Red-conjugated phalloidin. Images were acquired with a Zeiss LSM 880 confocal microscope and analyzed with Image J.

*Endocytosis assays-* For the integrin trafficking assay, cells were cultured on coated coverslips in 24-well plates overnight, after which they were treated with DMSO or Vacuolin-1 (1 μM) for 6 h. The cells were then placed on ice for a few minutes, and they were sequentially incubated with an anti-integrin primary antibody and an Alexa Fluor 488-tagged secondary antibody. Each antibody incubation was performed on ice for 1.5 h. The cells were then incubated with DMEM containing 10% FBS at 37°C in a CO_2_ incubator, and they were fixed at the time points indicated, for further analysis.

For the transferrin trafficking assay, cells were seeded on coverslips in 24-well plates for 24 h, after which they were pretreated with DMSO or Vacuolin-1(1 μM) for 6 h. The cells then were incubated with transferrin-488 on ice for 1.5 h, and then they were incubated in DMEM with 10% FBS at 37°C in a CO_2_ incubator before being fixed at the time points indicated, for further analysis.

*Immunofluorescence staining-* Fixed cells were permeabilized with 0.1% Triton X-100 in PBS for 15 min. After washing with PBS, the cells were blocked with 5% bovine serum albumin for 1 h at room temperature followed by incubation with the indicated primary antibodies at 4°C overnight. The cells were washed again and incubated with the appropriate fluorescence-conjugated secondary antibody for 1 h at room temperature. After another round of washing, the cells were mounted with ProLong™ Diamond Antifade mountant (Thermo Fisher, P36970). Images were acquired with the Zeiss LSM 880 confocal microscope and then analyzed with the ZEISS ZEN microscopy software.

*Immunohistology-* The tissues or organs isolated from mice were fixed with 10% neutral buffered formalin, after which they were subjected to paraffin-embedded sectioning. In brief, the tissues were dehydrated sequentially in graded ethanol (i.e., 70%, 80%, and 95%) for 1 h each, followed by 100% ethanol for 1 h, and then the ethanol was cleared by immersion of the tissues in xylene for 1 h. The tissues were immersed in paraffin for 1 h and then were sectioned at 5μm with a microtome. The sections were deparaffinized in xylene for 5 min and rehydrated by sequential incubation in graded ethanol (100%, 100%, 95%, 80%, 70%, and 50%, each for 5 min). For hematoxylin-eosin (H&E) staining, sections were stained with hematoxylin for 10 min, washed with distilled water for 1 min and with acid alcohol for 15 s. The sections were then rinsed with distilled water for 10 min and stained with eosin for 3 min, after which they were washed with 100% ethanol for 5 min with three repeats followed by xylene for 5 min, and they were finally mounted for microscopic analysis. For immunofluorescence staining, sections after rehydrating were heated at 95°C in the antigen retrieval buffer (10 mM Citric Acid, 0.05% Tween 20, pH 6.0). Immunostainings were then performed as described above. 4-5 sections per sample in different positions were used for quantification.

*Pull-down assay and mass spectrum analysis-* Biotin or biotin-V1 was conjugated with magnetic streptavidin beads (Invitrogen, 65602), followed by incubation with HeLa cell lysates at 4°C overnight. The bead-bound proteins were washed three times with wash buffer (150 mM NaCl, 0.1% NP-40 and 25 mM Tris-HCl, pH 7.4) and then centrifuged. After centrifugation, the beads were equilibrated in 50 mM ammonium bicarbonate (pH 8.0) and then treated with trypsin (500 ng/ml) at 37°C for 4 h. They were then centrifuged, and the supernatants were collected and incubated again with trypsin (250 ng/ml) at 37°C overnight. This digestion was stopped by the addition of formic acid, and the samples were vacuum dried before being analyzed by mass spectrometry.

*RAB5A-GTP activity -* RAB5A-GTP activity was determined by using the GST-R5BD pulldown assay . Cells were lysed in lysis buffer (25 mM HEPES-KOH (pH7.4), 100 mM NaCl, 5 mM MgCl2, 0.1 % NP40, 10 % Glycerol, 1 mM DTT, 2 μM leupeptin, 2.5μg/ml trypsin inhibitor, 0.1 mM PMSF and 2μg/ml aprotinin) containing 1% Triton X-100 at 4 °C for 5 min followed by centrifugation at 10,000 x g for 10 min. Equal amounts of protein were then added to the glutathione-Sepharose beads (GE Healthcare Biosciences) coated with 20 μg of the GST-fusion protein with the RAB5 binding domain from RABEP1 (simply referred to as GST-R5BD hereafter) and incubated at 4 °C for 60 min. The beads were washed four times with lysis buffer without detergent and boiled in SDS-sample buffer for elution. GTP-RAB5A proteins bound to the beads were analyzed by immunoblotting with an anti-RAB5A antibody.

*CRISPR/Cas9 genomic editing-* sgRNA sequences were designed by CHOPCHOP (Nucleic Acids Res. 42, W401–7 (2014). CapZβ-KO clonal cell lines were then generated by infection with lentiCRISPRv2 containing CapZβ-targeting sgRNA sequences (5’-GGAGATCCTCA CATAGACTG-3’ for human CapZβ, or 5’-CACCGACTGCGCCTTGGACCTGATG-3’ for mouse CapZβ), followed by single-cell cloning and puromycin selection. The knockout efficiency was validated by immunoblot analysis.

*Animal studies-* BALB/c athymic nude mice and breast cancer transgenic mice (FVB/N-Tg(MMTV-PyVT)634Mul/J) were purchased from the Jackson Laboratory (USA) and maintained in pathogen-free conditions with a 12 h light/dark cycle. All animal studies were performed according to the guidelines approved by the Animal Ethics Committee of City University of Hong Kong.

For the orthotopic metastasis model, 4T1 cells (1 × 10^5^) were injected into the right fourth mammary fat pad of 8-week-old female BALB/c athymic nude mice. When the tumor reached approximately 5 mm in diameter, the mice were received either the vehicle control (PEG400/Ethanol/tween80, 1:1:1) or V1 either orally or via IP every day. The primary tumor size (measured with calipers) and total body weight were determined every 5 days. At the endpoint, the mice were euthanized and the lungs were injected intratracheally with India ink and fixed in Fekete’s solution. The amount of metastasis was quantified by counting the number of white tumor nodules on the lung surface.

For the experimental metastasis model, 4T07-Luciferase-mcherry cells (1.5 × 10^5^) were intravenously introduced into 8-week-old female BALB/c athymic nude mice. The mice then received V1 or the vehicle control either orally or via IP every day. Luminescent images of the whole body were taken every 2-3 days using the IVIS Lumina III System (Caliper nLife Sciences) following IP injection of 150 mg/kg D-luciferin (Promega). At the endpoint, mice were euthanized and the lungs and other major organs were excised and imaged for luminescence. All luminescent images were acquired and analyzed using the Living Image software (Caliper Life Sciences).

MMTV-PyVT transgenic mice were verified by genotyping. Four-week-old female transgenic mice received V1 or the vehicle control orally or via IP every day. When the mice reached 110 days old, they were euthanized and the lungs were excised for H&E staining. The number of tumor nodules in the lung, as well as the tumor weight and the number of mammary tumors per mouse were quantified.

*Statistical analysis-* The data were presented as mean ± s.e.m., and the statistical significance of differences was determined by the unpaired Student’s t-test or ANOVA test. The asterisks indicate significance values P < 0.05 (*), P < 0.01 (**) and P < 0.001 (***); whereas P > 0.05 was considered to be not significantly different.

**Supplemental Results**


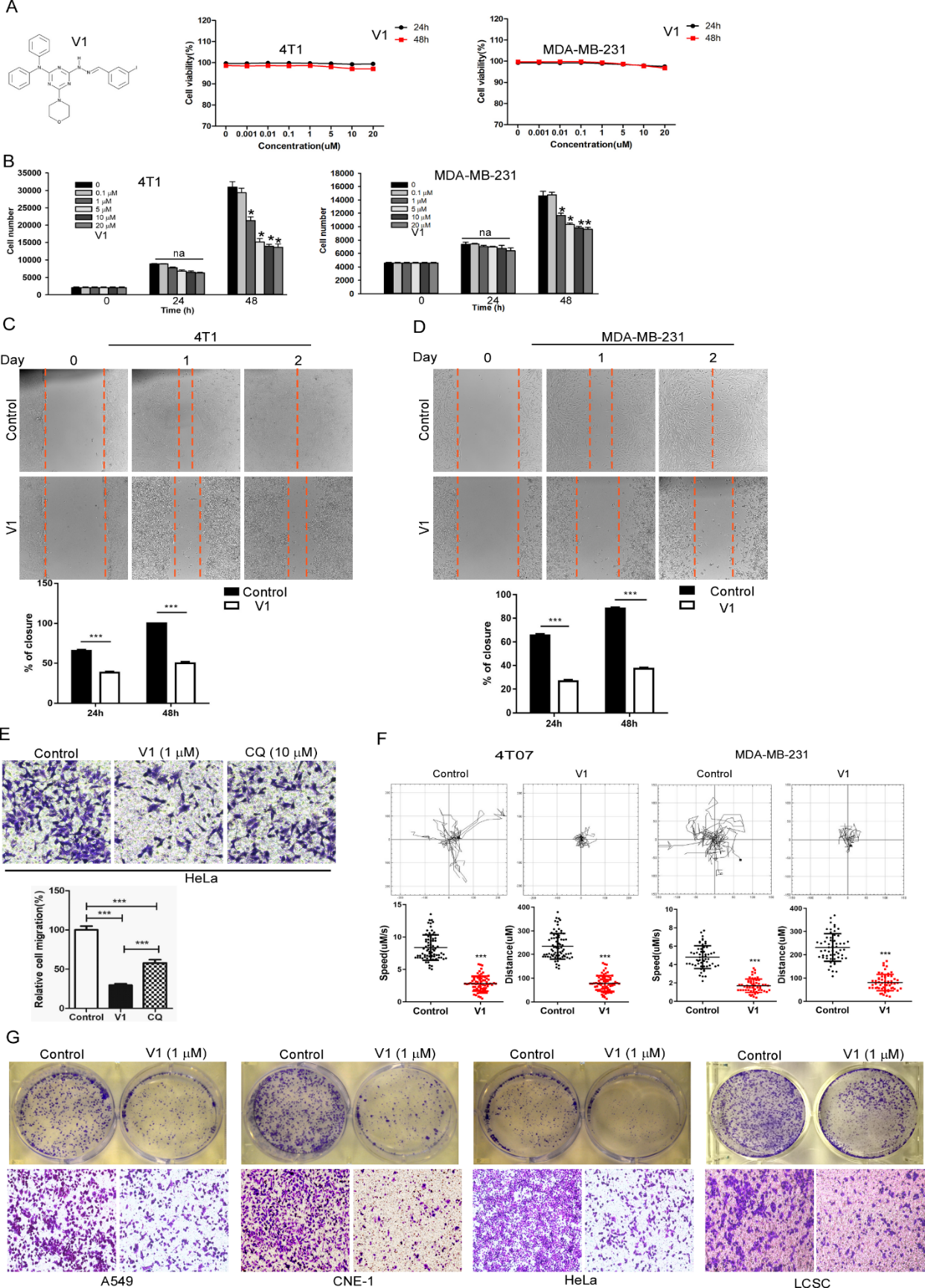


**Figure S1. V1 significantly inhibits cell migration and has low cell toxicity. (A, B)** 4T1 or MDA-MB-231 were placed at 96-well plates in triplicate and treated with or without V1 at the concentrations indicated for 24 h or 48 h. The cell viability was quantified via the MTT assay (**A**) or cell number was counted (**B**). (**C, D**) 4T1 (**C**) or MDA-MB-231 (**D**) cells were plated in 24-well plates, treated with or without V1 (1 μM), and then scratch-wounded. The borders of the scratch-wounds were imaged and the percentage of closure was quantified at 0, 24, and 48 h post-scratching. (**E**) HeLa cells were seeded into the upper chamber of transwell plates in the presence or absence of V1 or chloroquine (CQ) at the indicated concentrations, and after 18 h, the cells in the lower chambers were stained with crystal violet. (**F**) 4T07 or MDA-MB-231 cells were plated in 6-well plates and treated with or without V1 (1 μM) for 1 h followed by live-cell imaging to track the cell movement for 12 h at 30 min intervals. The distance and speed of cell movement were quantified. (**G**) V1 (1 μM) significantly inhibited the migration and colony formation of A549, CNE-1, HeLa, and human lung cancer stem cells (LCSCs) cells*.* The graphs represented data from three independent experiments, and data qualifications were expressed as mean ± s.e.m, *P < 0.05, ** P < 0.01, ***P < 0.001.


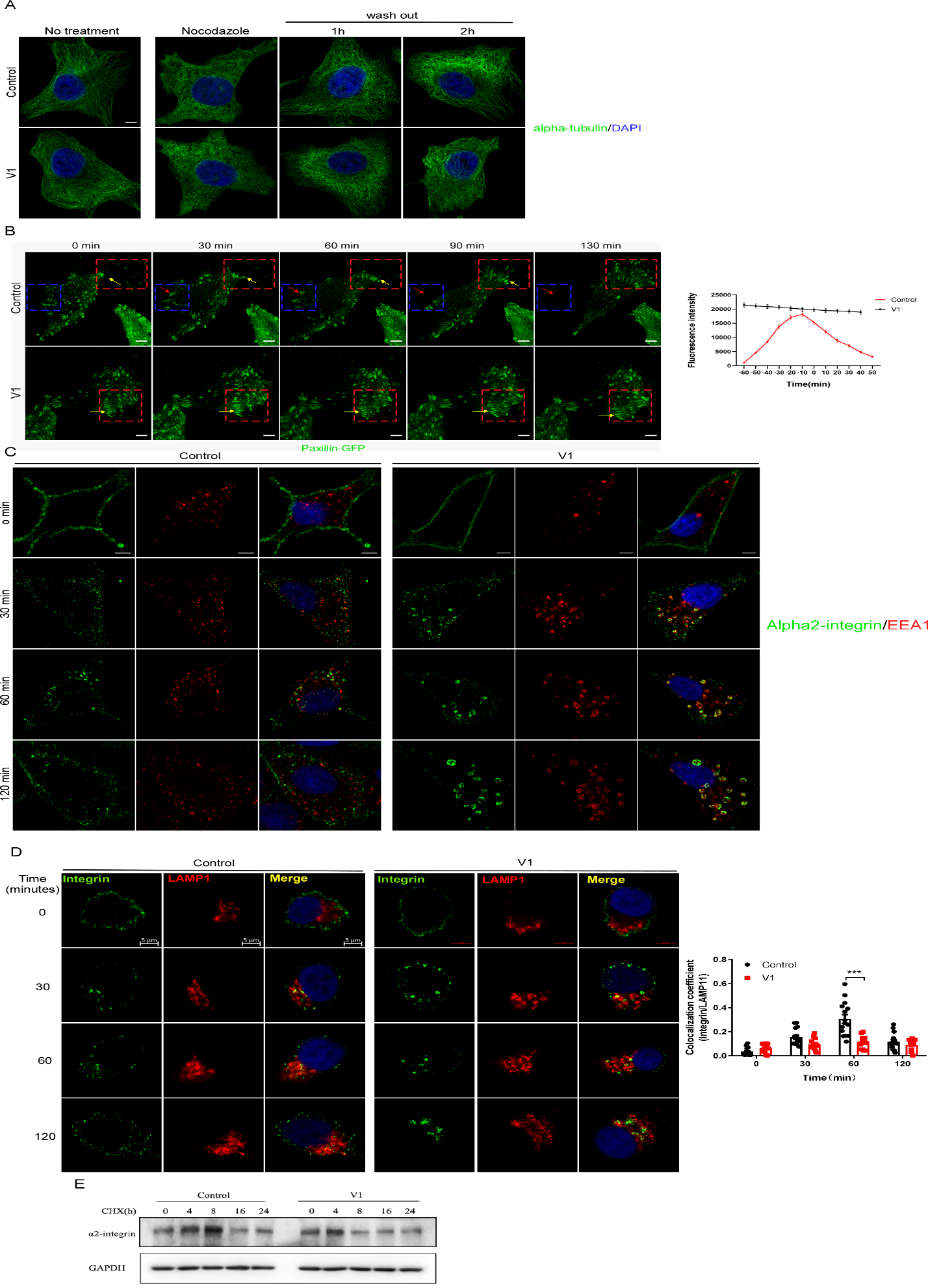


**Figure S2. V1 inhibits the recycling of integrins. (A)** HeLa cells were plated on coverslips in 24-well plates and treated with nocodazole (NOC) in the absence (control) or presence of V1 (1 μM). At indicated time points after removing NOC, the cells were stained with an anti-a-tubulin antibody. The scale bar is 10 μm. (**B**) Live GFP–paxillin-expressing HeLa cells treated with or without V1 (1μM) were imaged using a confocal microscope in a 10-min interval for 130min. The selected frames of the time-lapse movies are presented here. The fluorescence intensity of each FA spot in the cell, as shown by the arrows, is tracked and calculated. The curve between the fluorescence intensity of FA spot and time (the highest fluorescence intensity is designated as time 0) was plotted. 7-10 spots are selected to quantify in each cell. **(C)** A549 cells were plated on coverslips in 24-well plates and treated with or without V1 (1 μM), followed by incubation with an anti-α2 integrin antibody on ice for 1.5 h. At the indicated time points after release from cold arrest, the cells were fixed and stained with an anti-EEA1 antibody. Scale bar is 5μm. (**D**) HeLa cells were plated on coverslips in 24-well plates and treated with or without V1 (1 μM), followed by incubation with an anti-α2 integrin antibody on ice for 1.5 h. At the indicated time points after release from cold arrest, cells were immunostained with an anti-LAMP1 antibody. Scale bar is 5μm. (**E**) HeLa cells in the presence of cycloheximide were treated with V1 (1 μM) for the indicated times, followed by integrin immunoblot analysis.


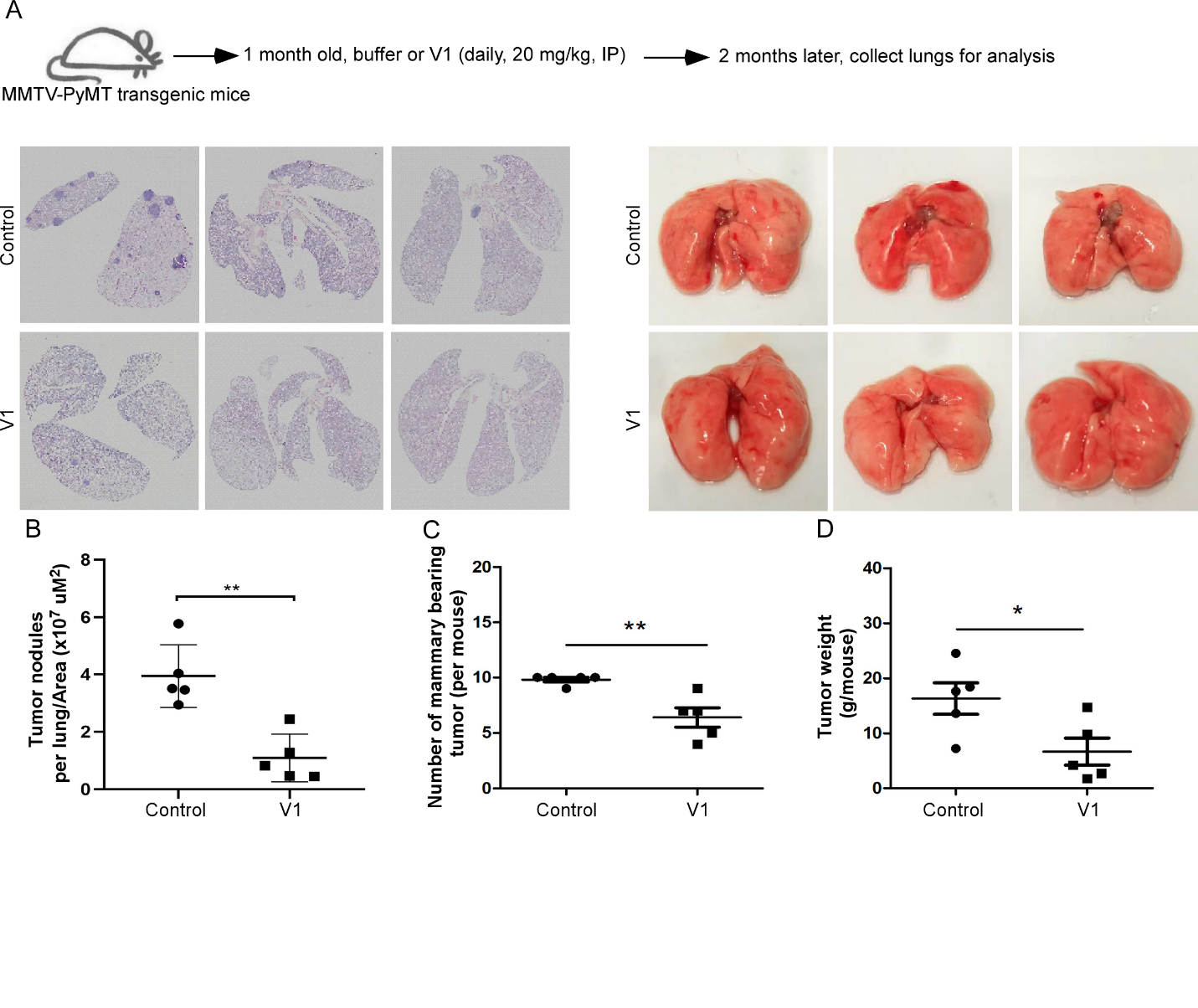


**Figure S3. V1 significantly inhibits mammary carcinoma metastasis in the MMTV-PyMT transgenic mouse model.** One-month-old female MMTV-PyMT transgenic mice were randomly divided into three groups (n=5 per group) and treated with either buffer or V1 (20 mg/kg, IP, daily) for 2 months. At the end of the experiment, the lungs in each group were collected and subjected to H&E staining (**A**), and the number of tumor nodules in each pair of lungs (**B**), the number of mammary bearing tumors (**C**) and the weight of mammary gland tumors (**D**) were measured and quantified. Data qualifications were expressed as mean ± s.e.m, *P < 0.05, ** P < 0.01, ***P < 0.001..


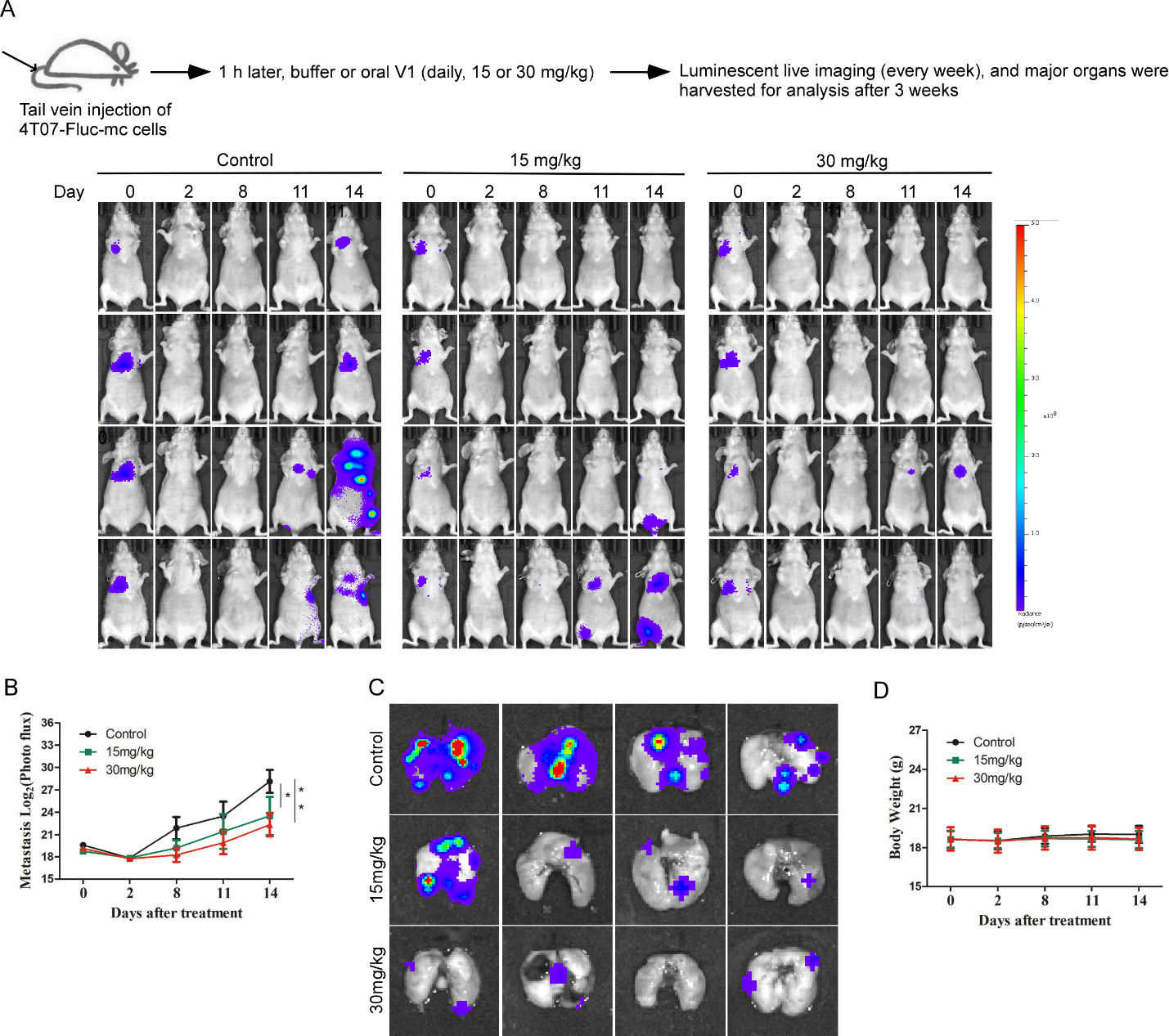


**Figure S4. V1 (oral) significantly inhibits tumor metastasis in an experimental mouse model.** Fluc-mCherry-expressing 4T07 cells were injected into the tail vein of nude mice. 1 h after injection, the mice were randomly divided into two groups (n=5 per group) and treated with either buffer or V1 (at either 15 or 30 mg/kg, daily) via an oral gauge for 2 weeks. During the course of the experiment, the mice were imaged every 3-4 days, and the luminescent signals were quantified (**A** and **B**). At the end of the experiment, the lungs (**C**) in each group were collected and imaged, and the luminescent signals were quantified. During the course of the experiment, the mouse body weight (**D**) were measured every 2~3 days. Data shown are mean ± s.e.m, *P < 0.05, ** P < 0.01, ***P < 0.001.


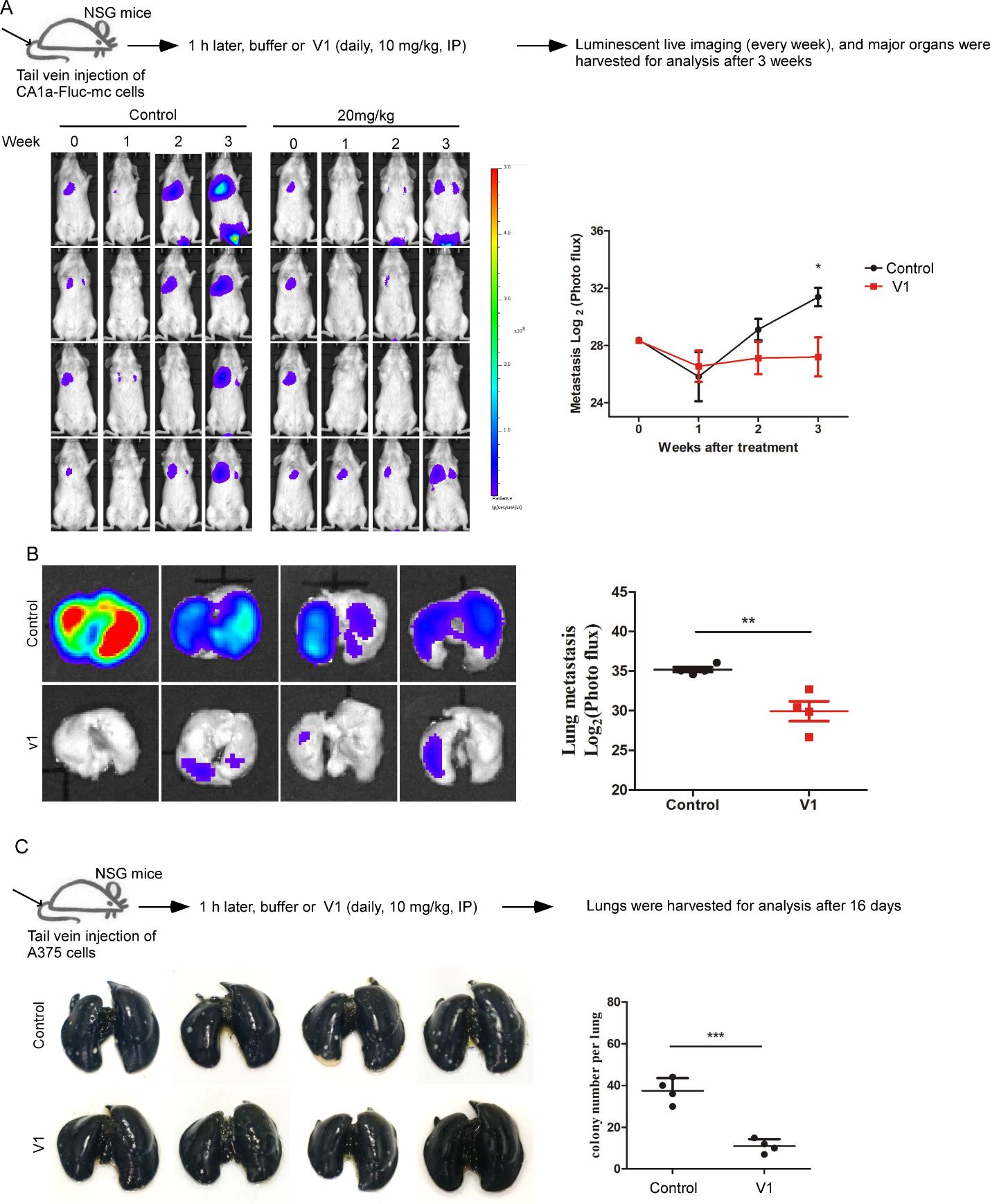


**Figure S5. V1 significantly inhibits the metastasis of human breast cancer or melanoma cancer in an experimental mouse model. (A)** and **(B)** Fluc-mCherry-expressing CA1a human breast cancer cells were injected into the tail vein of NSG mice. 1 h after injection, the mice were randomly divided into two groups (n=4 per group) and treated with either buffer or V1 (10 mg/kg, IP, daily) for 3 weeks. During the course of the experiment, the mice were imaged every week, and the luminescent signals were quantified (**A**). At the end of experiment, the lungs in each group were collected and imaged, and the luminescent signals were quantified (**B**). (**C**) A375SM human melanoma cancer cells were injected into the tail vein of NSG mice. 1 h after injection, the mice were randomly divided into two groups (n=4 per group) and treated with either buffer or V1 (10 mg/kg, IP, daily) for 16 days. At the end of experiment, the lungs in each group were collected and stained with ink, and the number of tumor nodules was quantified. Data qualifications were expressed as mean ± s.e.m, *p < 0.05, **p < 0.01, ***p < 0.001.


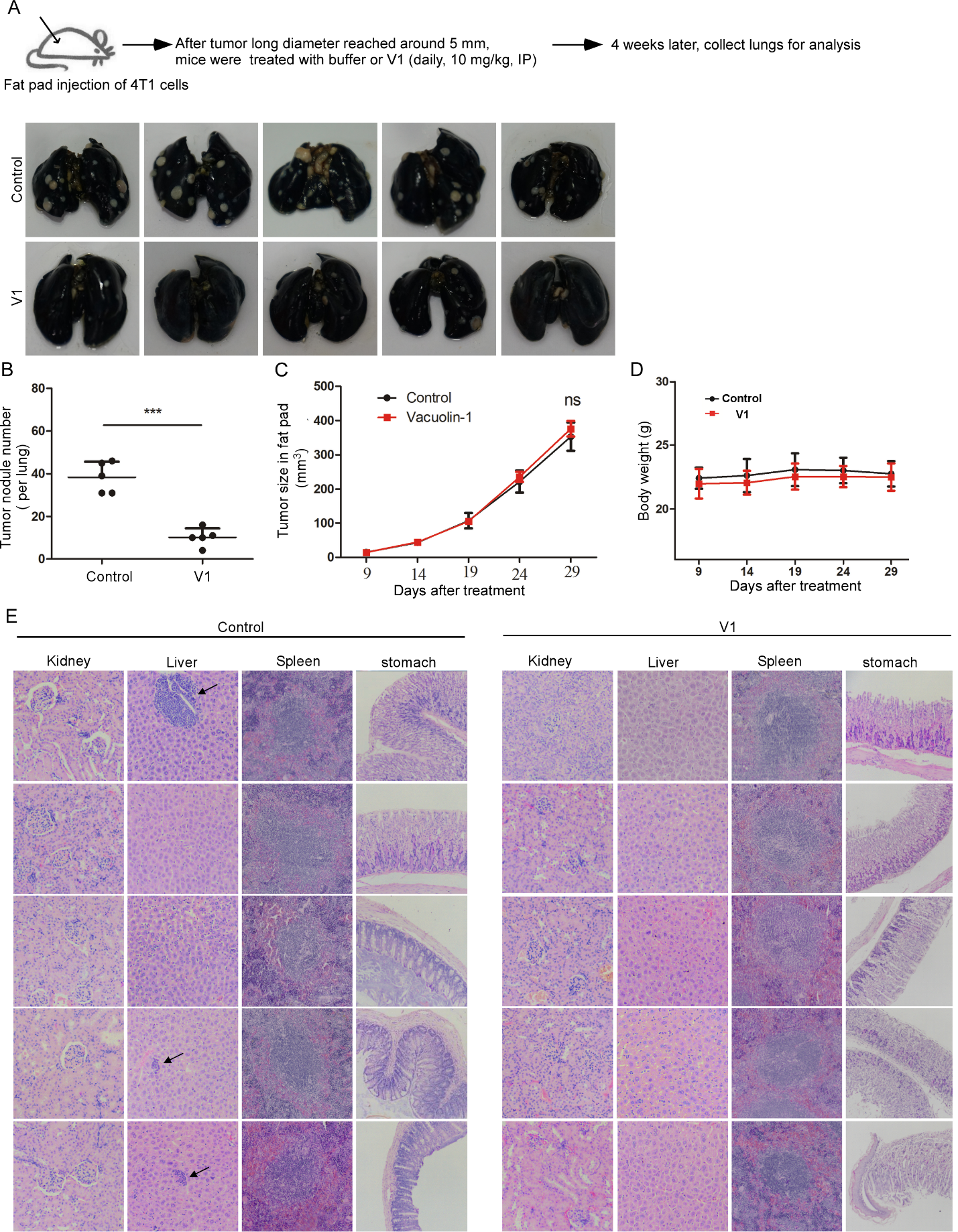


**Figure S6. V1 significantly inhibits tumor metastasis in an orthotopic metastatic mouse mammary carcinoma model.** 4T1 cells were injected into the fat pads of female nude mice. When the tumor was ~5 mm in long diameter, the mice were randomly divided into two groups (n=5 per group) and treated with either buffer or V1 (10 mg/kg, IP, daily) for 4 weeks. At the end of the experiment, lungs were collected for ink staining **(A)** and tumor nodules on the lung were measured **(B)**. During the experiment, tumor size **(C)** and mice body weight **(D)** were measured every 5 days. The major organs in each group (i.e., the kidney, liver, spleen and stomach) were collected and subjected to H&E staining (**E**). Data qualifications were expressed as mean ± s.e.m, *p < 0.05, **p < 0.01, ***p < 0.001.


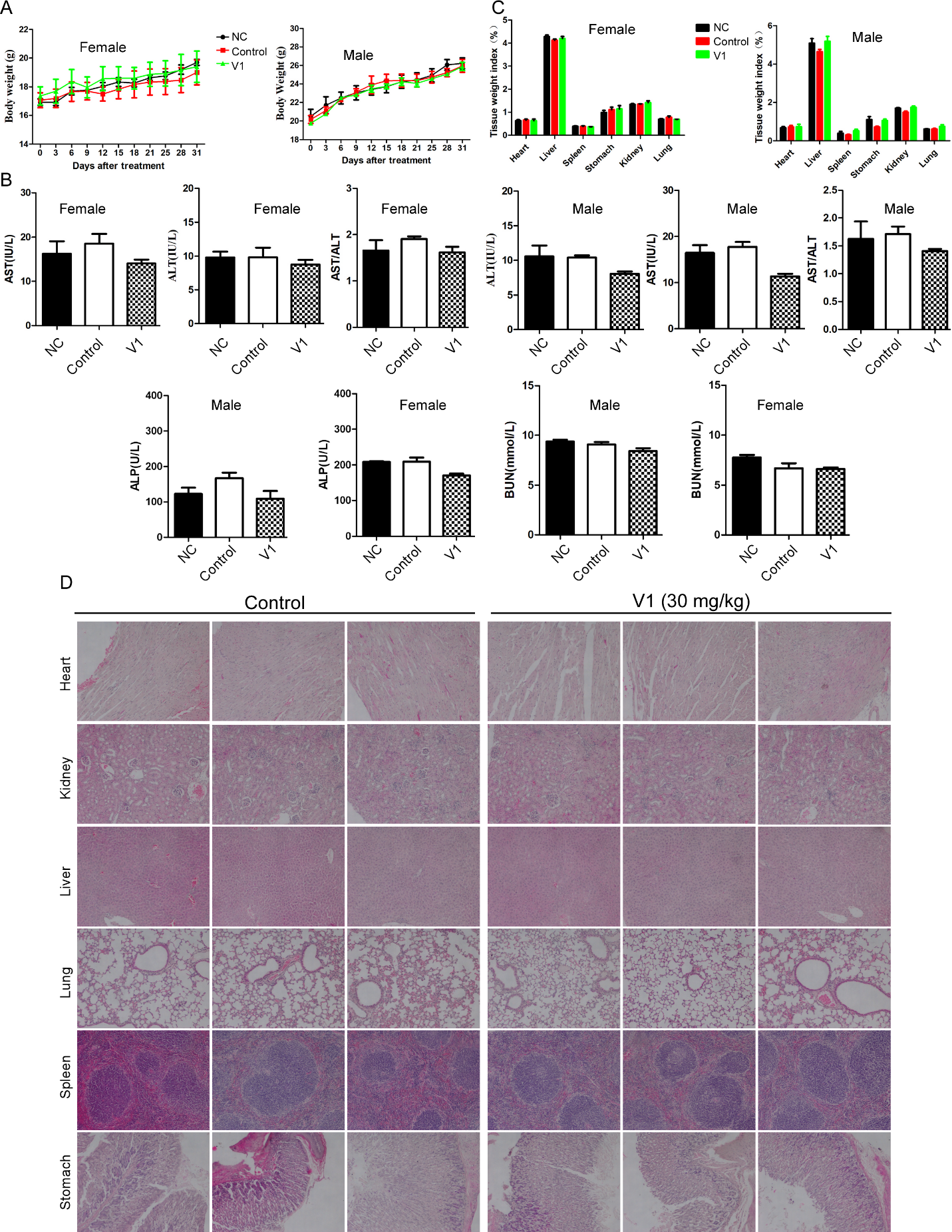


**Figure S7. The subchronic toxicity of V1 in mice.** 6 weeks old female and male Balb/C mice were given water (NC), vesicle control (PEG400/Ethanol/tween80, 1:1:1), or V1 (30 mg/kg, per day) via oral route for 30 days. (**A**) The body weight of female or male mice in each group were measured every three days during the course of drug treatment. (**B**)-(**D**) At the end of experiments, the bloods were collected from mice in each group for AST, ALT, ALP, and BUN measurement (**B**), and the mice were then sacrificed, the major organs were isolated, examined, weighted (**C**), sectioned, and subjected to H&E staining (**D**).


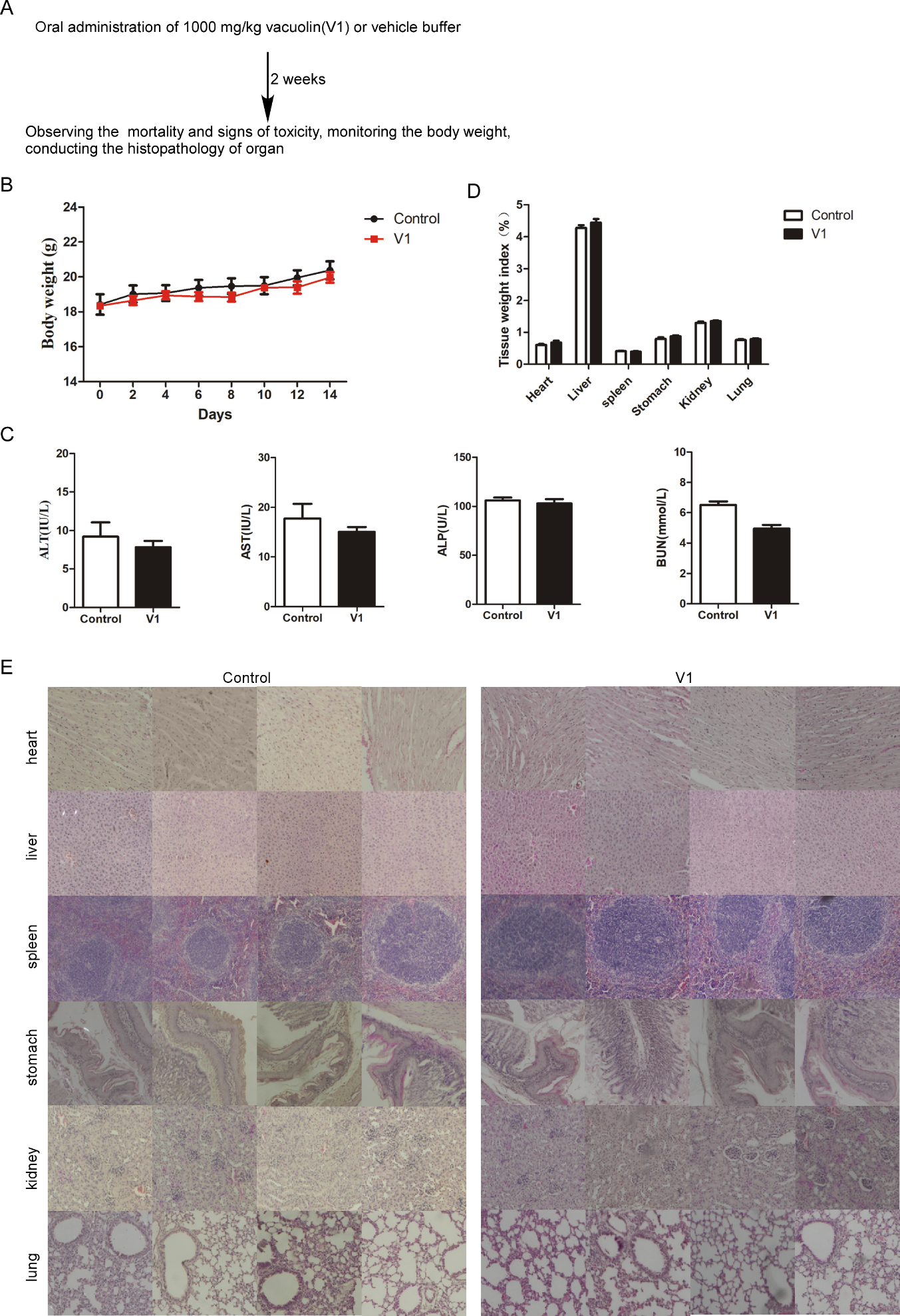


**Figure S8. The acute toxicity of V1 in mice.** 6 weeks old female Balb/C mice were given vesicle control or V1 (1000 mg/kg, one time) via oral route. (**A, B**) The body weight of female or male mice in each group were measured every three days during the course of drug treatment. (**C**)-(**E**) At the end of experiments, the bloods were collected from mice in each group for AST, ALT, ALP, and BUN measurement (**C**), and the mice were then sacrificed, the major organs were isolated, examined, weighted (**D**), sectioned, and subjected to H&E staining (**E**).

**
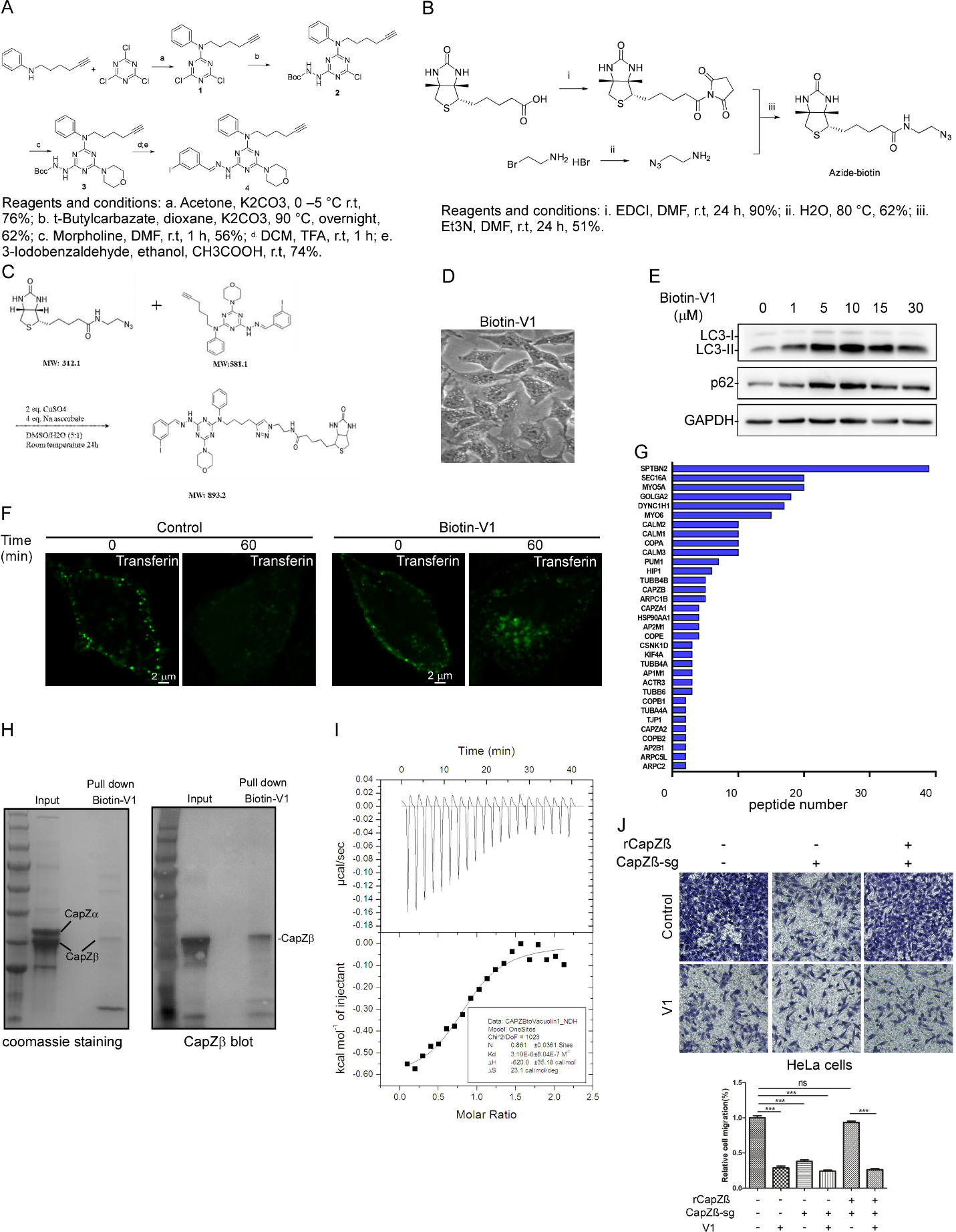
**

**Figure S9. Identification of CapZβ as a V1 binding protein. (A)** The synthesis route and conditions of a V1 analogue carrying a clickable terminal (alkynyl group). (**B**) The synthesis route and conditions of azide-biotin. (**C**) The generation of biotin-V1 by the click reaction. (**D**) and (**E**) Biotin-V1 induced large vacuoles (**D**), induced the accumulation of LC3-II and p62 (**E**), and inhibited transferrin degradation (**F**) in HeLa cells. (**G**) 33 of the proteins identified in the biotin-V1 complex are clustered in the endosomal trafficking pathway; the numbers indicate the number of peptides in each protein. (**H**) Purified His_6_-CapZα and His_6_-CapZβ recombinant proteins were incubated with biotin or biotin-V1, followed by streptavidin pulldown, and the streptavidin pulldowns were subjected to CapZβ immunoblotting or coomassie blue staining. (**I**) The interaction between biotin-V1 and CapZβ was assessed by isothermal titration calorimetry (ITC). (**J**) CapZβ knockout inhibited the migration of HeLa cells, which was restored by addback of rCapZβ. V1 treatment had no additive effect on the inhibition of migration of the CapZβ knockout cells.


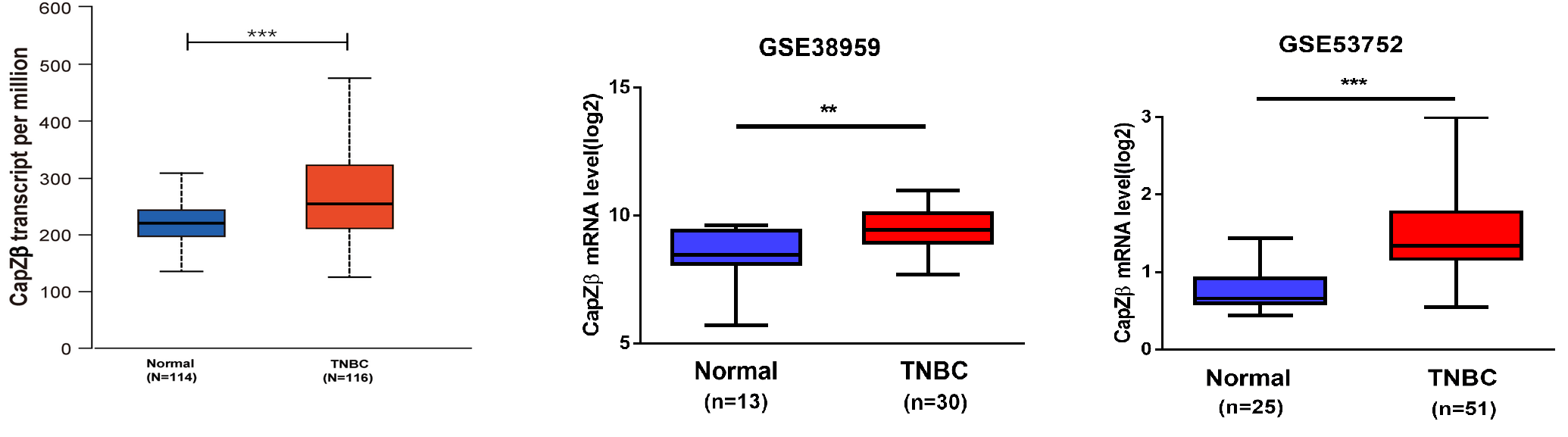


**Figures S10. CapZβ mRNA expression in TNBC**. **CapZβ expression in TNBC dataset of TCGA was analyzed via UALCAN. The datasets of GSE38959 and GSE53752 were downloaded from the Gene Expression Omnibus (GEO) database, and CapZβ mRNA level between normal and TNBC tissues was analyzed.**

**Video S1 and S2.** Live GFP–paxillin-expressing HeLa cells treated with or without V1 (1μM) were imaged using a confocal microscope in a 10-min interval for 130 min. Cells were maintained at 37 °C and 5% CO2 using a stage-top incubator.
