# Supplementary material for: Vacuolin-1 inhibits endosomal trafficking and metastasis via CapZβ
Source: Oncogene. 2021 Feb 9;40(10):1775–91. doi: 10.1038/s41388-021-01662-3 (PMC7946642; doi:10.1038/s41388-021-01662-3)
Supplement: Supplementary file 5 — author change form 2 [file 41388_2021_1662_MOESM5_ESM.pdf]

SPRINGER NATURE

In accordance with Springer Nature Authorship Policy we agree to change the authors of the manuscript as indicated below.

NAME OF JOURNAL: Oncogene

TITLE OF MANUSCRIPT: Vacuolin-1 inhibits endosomal trafficking and metastasis via CapZβ

MANUSCRIPT NUMBER: ONC-2020-01275

CORRESPONDING AUTHORS NAME: Yue Jianbo

PREVIOUS AUTHOR NAMES:

Zuodong Ye, Dawei Wang, Yunjiao He, Jingting Yu, Yingying Lu, Wenjie Wei, Chang Chen, Liangren Zhang, Hongmin Zhang, Jianbo Yue

UPDATED AUTHOR NAMES:

Zuodong Ye, Dawei Wang, Yingying Lu, Yunjiao He, Jingting Yu, Wenjie Wei, Chang Chen, Rui Wang, Minh Le, Liang Zhang,Liangren Zhang, William C. Cho,Mengsu Yang, Hongmin Zhang, Jianbo Yue

CHANGE TO AUTHOR LIST:

add Rui Wang, Liang Zhang, Minh Le, William C. Cho, Mengsu Yang

Print Name	Signature	Date
Minh Le	[signature]	13/11/2020
Liang Zhang	[signature]	29/09/2020
Liangren Zhang	[signature]	29/09/2020
William C. Cho	[signature]	28/09/2020
Mengsu Yang	[signature]	21/12/2020
Hongmin Zhang	[signature]	29/09/2020
Jianbo Yue	[signature]	29/09/2020
